# Self‐Powered α Radionuclide Nanomedicine: Mitochondria‐Targeted Multimodal Energy Recycling for Amplified Radioimmunotherapy

**DOI:** 10.1002/adma.202504612

**Published:** 2025-07-02

**Authors:** Xian Li, Chaochao Wang, Yelin Wu, Jiajia Zhang, Han Zhang, Shanshan Qin, Linglin Tang, Fei Yu

**Affiliations:** ^1^ Department of Nuclear Medicine, Shanghai Tenth People's Hospital Tongji University School of Medicine Shanghai 200072 P. R. China; ^2^ Institute of Nuclear Medicine Tongji University School of Medicine Shanghai 200072 P. R. China; ^3^ Department of Medical Ultrasound, Shanghai Tenth People's Hospital School of Medicine, Tongji University Shanghai 200072 P. R. China; ^4^ Department of Nuclear Medicine, Renji Hospital, School of Medicine Shanghai Jiao Tong University No. 160 Pujian Road Shanghai 200127 China

**Keywords:** alpha therapy, mitochondria‐targeted, radiodynamic, radionuclide therapy, radium‐223

## Abstract

Internal Radionuclide Therapy (IRT) faces significant challenges, particularly the limited controlled penetration depth of conventional β rays and the inefficient targeted delivery of α‐emitters. In this study, a mitochondria‐targeted, self‐powered α radionuclide nanomedicine, and pioneer a groundbreaking “suborganelle precise radiodynamic immunotherapy” paradigm that synergistically integrates physical irradiation, catalytic chemistry, and immunomodulation to overcome the historical limitations of IRT is developed. The innovation establishes a “radionuclide energy internal cycling” strategy through ^223^RaCl_2_ (the first FDA‐approved α‐emitter), unlocking three synergistic therapies from one radionuclide: precise ionizing radiation, self‐powered catalysis, and immunogenic reprogramming. This paradigm uniquely exploits the full decay spectrum (α particles, β electrons, γ photons) to synchronize physical, chemical, and biological anti‐tumor mechanisms without requiring external energy inputs, offering a transformative solution to overcome the physical‐biological barriers of IRT and bridge localized eradication with systemic immune regulation.

## Graphical abstract

A multifunctional nanomedicine co‐loading of the α‐emitter ^223^RaCl_2_ within iron‐based MOFs is developed. Precisely targeted to mitochondria, it exploits the full decay spectrum to synchronize three therapeutic actions: direct α‐particle ionization, self‐powered catalytic H_2_O_2_ generation via secondary electrons, and immunogenic cell death induction. This integrated approach overcomes the limitations of conventional radiotherapy by facilitating effective local tumor ablation and systemic anti‐tumor immunity without external energy input.

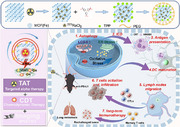

## 1 Introduction

Internal Radionuclide Therapy (IRT) remains indispensable for the ablation of solid tumors. Traditional β rays face inherent limitations due to their uncontrolled penetration depth, resulting in poor efficacy and collateral damage to normal tissue.^[^ ^1^ ^]^ Targeted Alpha therapy (TAT) emerges as a promising therapeutic modality owing to its characteristically high linear energy transfer (LET, 80–100 keV µm^−1^) and confined range of radionuclides (50–100 µm), enabling precise energy deposition within tumor microenvironments.^[^ ^2^, ^3^ ^]^ Nevertheless, clinical translation of TAT confronts some challenges: inherent instability of chelation, absence of subcellular organelle targeting precision, and insufficient tumor accumulation efficiency.^[^ ^4^, ^5^, ^6^ ^]^ Failure to achieve accurate delivery to tumor‐specific subcellular compartments risks exacerbating dose‐limiting toxicities, thereby undermining therapeutic potential.^[^ ^7^ ^]^ Therefore, how to achieve precise delivery of α‐nuclides and synergistic activation of anti‐tumor immunity is an important challenge, and there is an urgent need to develop novel nuclide carriers to break through the physical‐biological barriers of traditional radiation therapy.^[^ ^8^ ^]^

Nanoscale metal‐organic frameworks (nMOFs) emerge as a paradigm‐shifting platform in precision radiotherapy, leveraging their tunable porosity architectures (enabling high payload capacity), inherent catalytic functionality, and versatile surface tailor ability to demonstrate distinctive advantages in therapeutic encapsulation, delivery and radiation potentiation— potentially presenting a promising solution to overcome the longstanding challenges in TAT.^[^ ^9^, ^10^, ^11^ ^]^ Currently, there are fewer reports on the interaction of α radionuclide with nMOF. And the existing studies are mainly confined to the single mode of action of conventional radiation therapy, i.e., relying only on ionizing damage and not utilizing the secondary energies (e.g., β‐particles, gamma‐photons) in the decay process.^[^ ^12^ ^]^ However, since some α radionuclide can emit multiple types of particles during their decay, such as the first α radionuclide currently approved by the FDA for clinical use, ^223^RaCl_2_, this oversimplified approach fundamentally limits therapeutic optimization and constitutes an underutilized biological effector. A paradigm shift toward multimodal energy utilization—synergistically integrating direct ionization, secondary particle interactions, and catalytically enhanced radiochemical processes—is necessary to fully harness the therapeutic potential of α radionuclide while mitigating systemic toxicity.

Mitochondria, the center of energy metabolism in eukaryotes, occupy a pivotal position in cell fate regulation and are also a key microenvironment for radiosensitization.^[^ ^4^, ^13^ ^]^ First, with the exception of the nucleus, mitochondria are the only organelles that contain DNA. Furthermore, Mitochondrial DNA (mtDNA) exhibits a higher sensitivity to ionizing radiation in comparison to nucleus DNA (nDNA).^[^ ^14^, ^15^ ^]^ This heightened sensitivity is attributed to the absence of a robust repair system in mtDNA, resulting in a significantly lower repair efficiency compared to nDNA.^[^ ^16^, ^17^ ^]^ Significantly, previous studies have demonstrated that death‐promoting mitochondrial autophagy triggers lysosomal membrane permeability, enhances tumor antigen presentation, induces immunogenic adenosine triphosphate (ATP) release, and activates anti‐tumor immunity.^[^ ^18^, ^19^ ^]^ Consequently, mitochondria emerge as a promising target for disrupting the immunosuppressive microenvironment and enhancing anti‐tumor efficiency. In view of this, the development of novel α radionuclide drugs that target mitochondria may prove a promising therapeutic strategy, achieving precise drug delivery and immune system activation to enhance anti‐tumor efficacy.

**Scheme 1:**
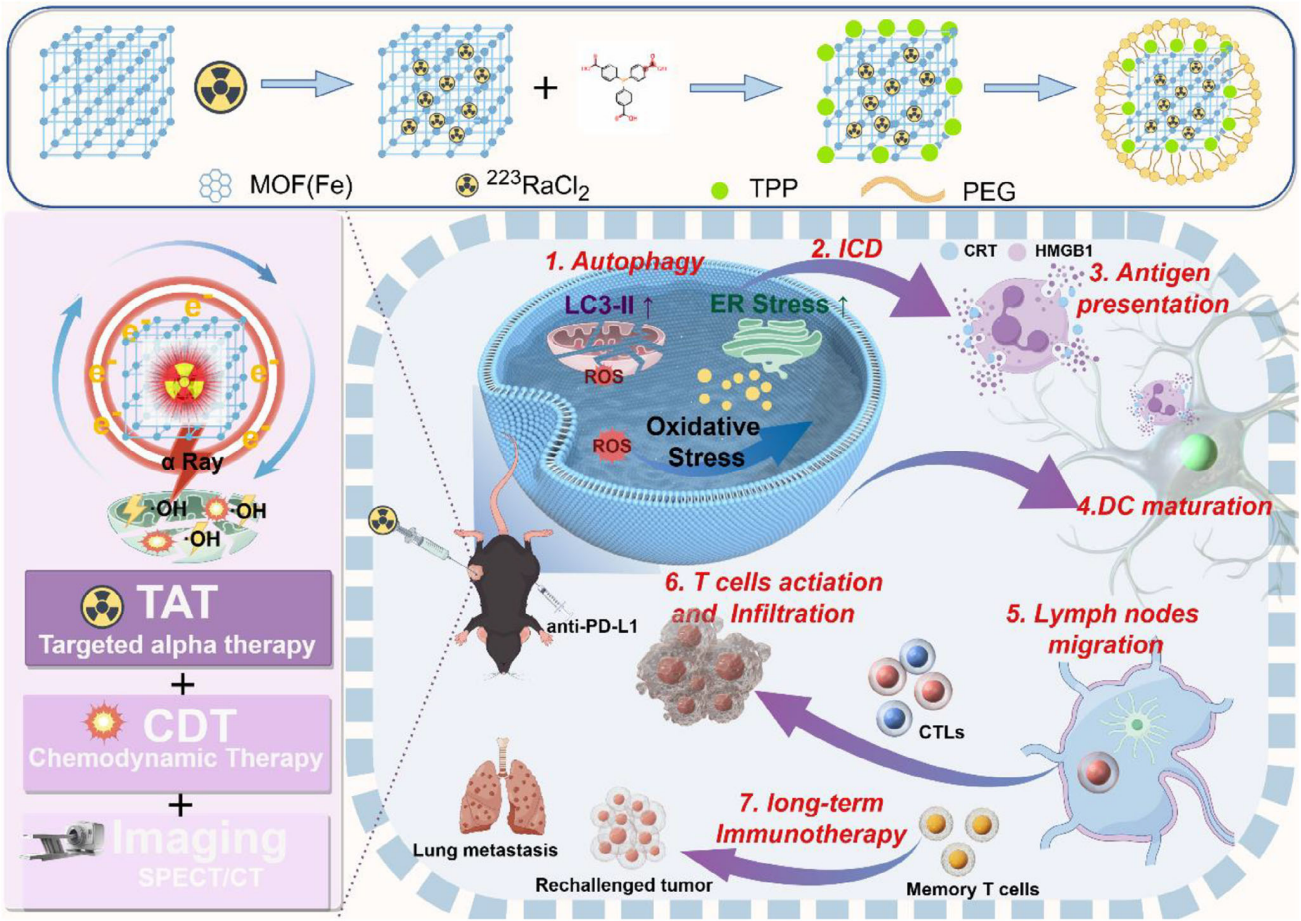
Illustration for the preparation of α nanoparticles and their mechanisms of multifunctional self‐powered for enhanced cancer therapy (By Figdraw).

This study pioneers a multifunctional self‐powered α radionuclide nanomedicine by strategically integrating the versatile decay properties of α radionuclide with nMOF technology (**Scheme** **1**). The engineered compound comprises four functional components: therapeutic α‐emitter ^223^RaCl_2_, iron‐based MOF matrices (MOF(Fe)), mitochondria‐targeting triphenylphosphonium groups and hydrophilic PEG coating. First, α particles trigger intensive ionization directly in the mitochondria by ionization, destroying mtDNA and respiratory chain complexes. Mitochondrial damage induces pro‐death mitochondrial autophagy further induces enhanced immunogenic death. Concurrently, the released secondary electrons act as an endogenous excitation source, promoting and accelerating the conversion of Fe^3+^ to Fe^2+^ in MOF(Fe) to enrich more H_2_O_2_ and enhance the Fenton reaction. This solves the critical bottleneck of conventional Chemodynamic Therapy (CDT) that relies on insufficient endogenous hydrogen peroxide (H_2_O_2_) (intracellular H_2_O_2_ in tumor cells is usually <100 µM). In addition, the released γ photon enables real‐time treatment monitoring through nuclear imaging. In a word, by capitalizing on mitochondria's inherent DNA repair deficiencies, this platform achieves irreversible physico‐chemical damage through three synergistic mechanisms: 1) direct damage to mitochondria by highly ionizing radiation from α particles, 2) self‐powered catalytic H_2_O_2_ generator from secondary electrons and 3) immunogenic cell death (ICD) potentiation. This breakthrough is attributable to the creation of an “intranuclear energy cycle” system, for the first time, enables a single radionuclide to activate energy channels in three dimensions — physical, chemical, and biological — to drive TAT, CDT, and immunotherapy concurrently, thereby effectively resolving the historical dichotomy between localized tumor treatment and comprehensive immune modulation.

Mitochondria‐targeting nanoplatforms constructed by MOF(Fe) co‐loading ^223^RaCl_2_ (α‐particles for TAT; secondary electrons boosting CDT; γ‐photons enabling SPECT) with concurrent TPP amidation, followed by PEG coating. The system drives reactive oxygen species (ROS) generation through combined α‐particle radiotherapy and secondary electron‐enhanced chemodynamics, prompting mitochondrial autophagy and ER stress to induce ICD (CRT/HMGB1 exposure). This initiates antitumor immunity: DCs mature, present antigens in lymph nodes, and activate CTLs; anti‐PD‐L1 enhances T‐cell infiltration, establishing durable immune memory against metastasis.

## 2 Results and Discussion

### 2.1 Synthesis and Characterization

**Figure 1:**
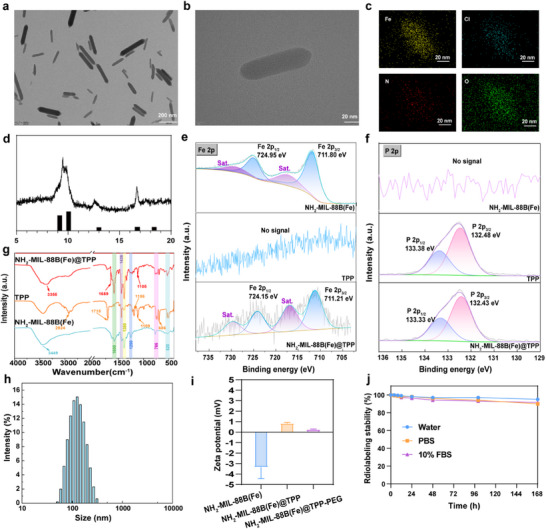
Characterizations of NH_2_‐MIL‐88B (Fe)@TPP‐PEG nanoparticles (NPs). a–c) TEM images of NH_2_‐MIL‐88B (Fe): a) Highly dispersed NH_2_‐MIL‐88 (Fe) nanocatalysts. Scale bar, 200 nm. b) A representative NH_2_‐MIL‐88B (Fe) NPs with a bipyramidal hexagonal prism morphology. Scale bar, 20 nm. c) EDS element mappings of Fe, Cl, N, and O of the indicated region in (b). Scale bar, 20 nm. d) Powder XRD pattern for the prepared NH_2_‐MIL‐88B (Fe) nanocrystal sample. XRD reflection for the Cr (III)‐based MIL‐88B structure based on CIF is presented as a reference. e,f) XPS spectra of NH_2_‐MIL‐88B (Fe), TPP and NH_2_‐MIL‐88B (Fe)@TPP. Fe 2p spectrum indicates that all the Fe components in NH_2_‐MIL‐88B (Fe) are in the valence of +3. g) FTIR spectra of NH_2_‐MIL‐88B (Fe), TPP and NH_2_‐MIL‐88B (Fe)@TPP. h) NH_2_‐MIL‐88B (Fe)@TPP‐PEG NPs diameter distribution. i) Zeta potential (mV) of NH_2_‐MIL‐88B (Fe), NH_2_‐MIL‐88B(Fe)@TPP and NH_2_‐MIL‐88B(Fe)@TPP‐PEG. j) and stability of ^223^Ra‐NH_2_‐MIL‐88B (Fe)@TPP‐ PEG in vitro (abbreviated as ^223^Ra‐MOF(Fe)@TPP).

Advanced structural characterization confirmed the successful synthesis and functionalization of the nanocomposite. High‐resolution transmission electron microscopy (TEM) and high‐angle annular dark‐field imaging revealed well‐dispersed biconical hexagonal prismatic nanostructures (**Figure** **1**a,b), demonstrating exceptional morphological uniformity with mean dimensions of 125.6 ± 3.2 nm in length and 34.2 ± 1.8 nm in width. Complementary energy‐dispersive X‐ray spectroscopy (EDS) elemental mapping (Figure 1c) verified the homogeneous spatial distribution of Fe, Cl, N, and O constituents, confirming compositional consistency across the nanoparticle matrix.

The crystalline architecture was validated through powder X‐ray diffraction (XRD) analysis, showing excellent phase matching with the MIL‐88B reference pattern (JCPDS 00‐066‐1395) (Figure 1d). The framework contains geometrically constrained secondary building units that generate abundant accessible Fe(III) catalytic centers,^[^ ^20^ ^]^ providing the basis for peroxidase‐mimetic catalytic functionality under acidic conditions. Subsequent X‐ray photoelectron spectroscopy (XPS) analysis (Figure 1e; Figure S1, Supporting Information) resolved the iron oxidation state, with the characteristic Fe 2p_3/2_ peak at 710.8 eV conclusively identifying trivalent iron species. This oxidation state proved critical for coordinating NH_2_‐BDC ligands and maintaining structural integrity through trimeric SBU formation. The strong mitochondrial targeting efficacy of TPP has been confirmed by many studies, and subsequently, the amino group of NH_2_‐MIL‐88B(Fe) (abbreviated as MOF(Fe)) amides with tris(4‐carboxyphenyl)phosphine oxide (a carboxyl‐functionalized TPP derivative, hereafter abbreviated as TPP in this work)t under the action of the activator (EDC/NHS) to form NH_2_‐MIL‐88B(Fe)@TPP (abbreviated as MOF(Fe)@TPP). Surface functionalization of NH_2_‐MIL‐88B(Fe)@TPP was systematically investigated through comparative spectroscopy. While the parent NH_2_‐MIL‐88B(Fe) exhibited exclusive Fe (III)‐O coordination features, the TPP‐modified counterpart displayed a distinct P 2p signal at 132.6 eV (Figure 1f), confirming successful phosphine group conjugation. Fourier‐transform infrared (FTIR) spectroscopy provided additional evidence of chemical modification: NH_2_‐MIL‐88B(Fe) showed signature vibrations at 3449 cm^−1^ (N─H stretch), 1578/1380 cm^−1^ (carboxylate antisymmetric/symmetric stretches), and 1250 cm^−1^ (C‐N vibration). After TPP modification, new absorption peaks appeared at 1105 cm^−1^ (P‐C aromatic ring stretching) and 686 cm^−1^, while the amino N‐H peaks (3300‐3500 cm^−1^) weakened, and new amide N─H (3355 cm^−1^) and C─N (1659 cm^−1^) peaks emerged (Figure 1g). Subsequent PEG conjugation enhanced biological compatibility while establishing a near‐neutral surface charge favorable for cellular internalization. To comprehensively track morphological evolution during functionalization, TEM analysis was extended to modified variants (Figure S2, Supporting Information): NH_2_‐MIL‐88B(Fe) presents uniform hexagonal/spindle nanoparticles with sharp edges and smooth surfaces. Upon TPP modification, NH_2_‐MIL‐88B(Fe)@TPP exhibits aggregation and surface roughness, consistent with hydrophobic TPP interactions. PEGylation of NH_2_‐MIL‐88B(Fe)@TPP‐PEG yields particles with blurred edges, confirming the formation of a hydrated PEG corona that reduces aggregation.

Hydrodynamic properties monitored by dynamic light scattering (DLS) revealed stepwise evolution: NH_2_‐MIL‐88B(Fe) (140.8 ± 5.6 nm; PDI 0.20 ± 0.05), NH_2_‐MIL‐88B(Fe)@TPP (151.4 ± 8.5 nm; PDI 0.28 ± 0.08, indicating minor aggregation), and NH_2_‐MIL‐88B(Fe)@TPP‐PEG (156.6 ± 7.1 nm; PDI 0.24 ± 0.04) (Figure 1h). Zeta potentials transitioned from −4.4 ± 0.1 mV (pristine) to +0.8 ± 0.12 mV (TPP‐modified, reflecting amino consumption) and finally +0.21 ± 0.04 mV (PEG‐conjugated, indicating charge shielding). (Figure 1i)

The final radiopharmaceutical construct demonstrated practical utility through efficient ^223^RaCl_2_ incorporation, achieving ≈80% radiochemical yield as quantified by paper chromatography. Remarkably, the ^223^Ra‐NH_2_‐MIL‐88B(Fe)@TPP‐PEG (abbreviated as ^223^Ra‐MOF(Fe)@TPP) conjugate maintained >90% radiochemical purity over 168 h (Figure 1j), indicating exceptional radionuclide retention capacity crucial for therapeutic applications. Long‐term colloidal stability assessments further confirmed robustness: MOF(Fe), MOF(Fe)@TPP, and MOF(Fe)@TPP‐PEG exhibited minimal hydrodynamic diameter changes over 5 days in water, PBS, and 10% FBS (Figure S3, Supporting Information). PEGylated variants showed superior stability (attributed to steric hindrance), while mild aggregation in PBS for non‐PEG samples was mitigated by overall structural integrity (Figure S4, Supporting Information).

Collectively, these multimodal characterization data validate the hierarchical design strategy combining structural precision, catalytic optimization, and biological compatibility in a single nanoplatforms.

### 2.2 Enhanced ·OH Yield in ^223^Ra‐MOF(Fe) NPs‐Mediated Fenton Reaction

**Figure 2:**
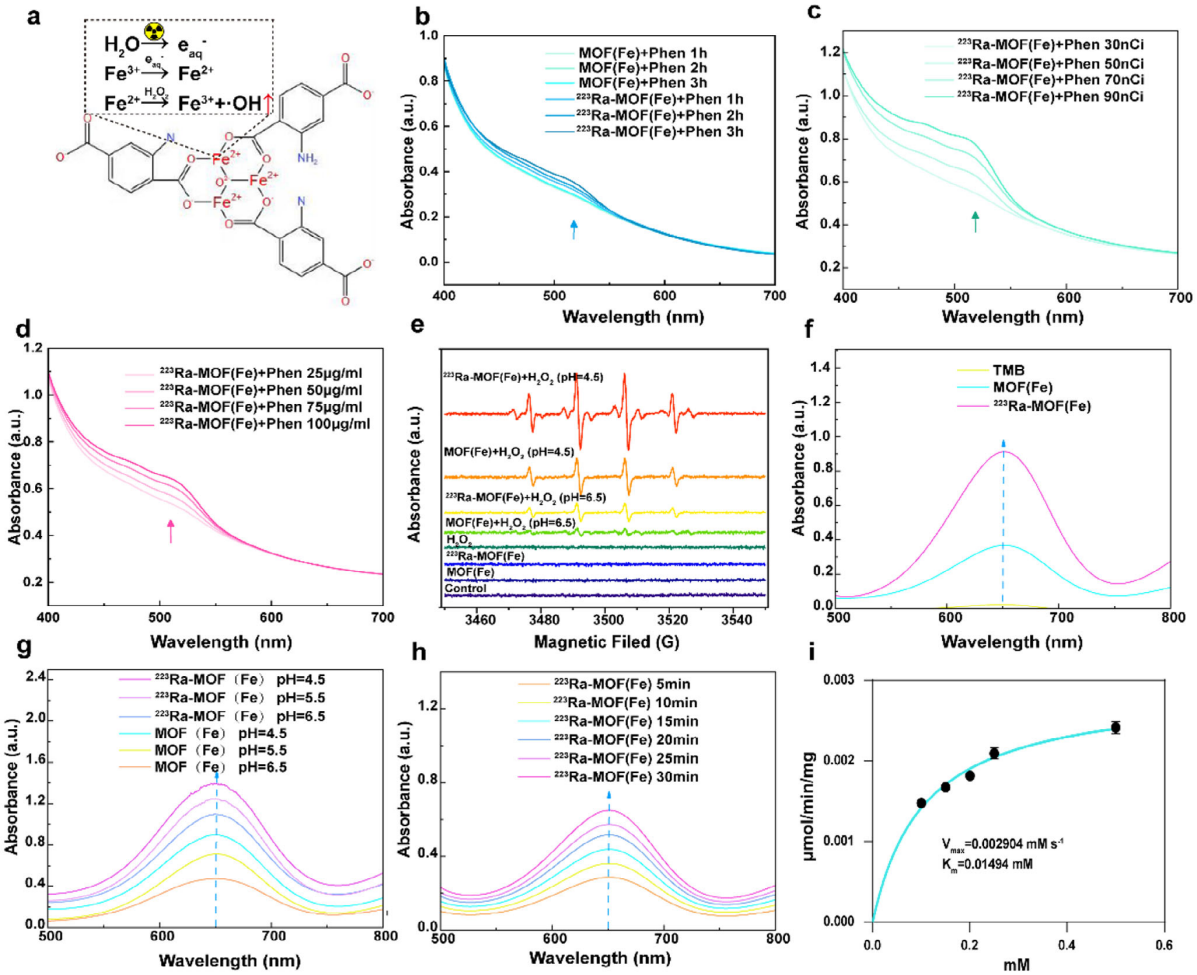
Enhanced ·OH yield in ^223^Ra‐MOF(Fe)@TPP NPs‐Mediated Fenton Reaction. a) The primary mechanism of ^223^Ra‐MOF(Fe) NPs possesses a high ·OH yield in the Fenton reaction. b) The absorptance spectrum of Phen solution containing the mixture of MOF(Fe) or ^223^Ra‐MOF(Fe)@TPP NPs. c) The absorptance spectrum of Phen solution containing the mixture of ^223^Ra‐MOF(Fe) under different doses. d) The absorptance spectrum of Phen solution containing the mixture of ^223^Ra‐MOF(Fe) under different concentrations. e) ·OH production in the indicated groups using ESR. f) POD‐like activity in different systems in the absence or presence of MOF(Fe) and ^223^Ra‐MOF(Fe. g) PH‐dependent POD‐like activity of MOF(Fe) and ^223^Ra‐MOF(Fe). h) Time‐dependent POD‐like activity of ^223^Ra‐MOF(Fe). i) Kinetic assay for the POD‐like activity of ^223^Ra‐MOF(Fe) with H_2_O_2_ as substrate. NH_2_‐MIL‐88B(Fe) (abbreviated as MOF(Fe)), NH_2_‐MIL‐88B(Fe)@TPP (abbreviated as MOF(Fe)@TPP), ^223^Ra‐NH_2_‐MIL‐88B(Fe) (abbreviated as ^223^Ra‐MOF(Fe)), ^223^Ra‐NH_2_‐MIL‐88B(Fe)@TPP‐PEG (abbreviated as ^223^Ra‐MOF(Fe)@TPP).

The Fenton reaction in ^223^Ra‐MOF(Fe) NPs involves secondary electron‐induced production of hydrated electrons (e_aq_⁻), accelerating Fe^3+^/Fe^2+^ conversion and enhancing ·OH accumulation (**Figure** **2**a). To distinguish between Fe^3+^ and Fe^2+^, we used 1,10‐phenanthroline (Phen), a complexing reagent that forms an orange‐red [Fe (Phen)_3_]^2+^ complex with Fe^2+^, detectable by its absorption peak at 510 nm. Upon adding Phen to ^223^Ra‐MOF(Fe), the solution turned orange‐red, and the 510 nm absorption peak intensified over time, demonstrating that ^223^RaCl_2_ enabled sustained Fe^3+^/Fe^2+^ conversion and Fe^2+^ accumulation, while the Phen‐added MOF(Fe) solution remained stable (Figure 2b). At the same time, the absorbance increased progressively with the dose of ^223^Ra (Figure 2c) and the concentration of the material (Figure 2d).

To directly detect ·OH generation, electron spin resonance (ESR) spectroscopy was employed. In a simulated TME solution, ESR signals of 5,5‐dimethyl‐1‐pyrroline‐N‐oxide (DMPO‐OH) confirmed ·OH production in the ^223^Ra‐MOF(Fe)‐mediated Fenton reaction. Distinct DMPO‐OH peaks appeared only in the presence of both H_2_O_2_ and MOF(Fe) NPs or ^223^Ra‐MOF(Fe) NPs. The peak intensity was higher at pH 4.5 than at pH 6.5, reflecting enhanced Fenton activity under acidic conditions. Notably, the ^223^Ra‐MOF(Fe) + H_2_O_2_ group exhibited stronger DMPO‐OH signals than the MOF(Fe) + H_2_O_2_ group, indicating greater ·OH production (Figure 2e).

To verify this, the present study was carried out to systematically characterize the peroxidase (POD)‐like activity of MOF(Fe), ^223^Ra‐MOF(Fe) nanocatalysts. In acidic buffer system at pH 4.5, 3, 3′,5, 5′ tetramethylbenzidine (TMB) was used as a chromogenic substrate, and 500 µM H_2_O_2_ was added to MOF(Fe) and ^223^Ra‐MOF(Fe) containing MOF(Fe) and ^223^RaCl_2_, respectively, and the reaction was carried out for 5 min to monitor the amount of ·OH production in real‐time using the oxidation chromogenic reaction of TMB. The color of TMB was oxidized to blue cation radicals and showed characteristic absorbance changes at 652 nm, which showed that the absorbance intensity of ^223^Ra‐MOF(Fe) was much higher than that of MOF(Fe), indicating that the introduction of ^223^Ra greatly enhanced the POD‐like enzyme activity of MOF(Fe) (Figure 2f; Figure S5, Supporting Information). The POD‐like activities of MOF(Fe) and ^223^Ra‐MOF(Fe) at different pH values were compared, and the absorbance intensities of ^223^Ra‐MOF(Fe) at 652 nm were all much higher than that of MOF(Fe), suggesting that the absorbance intensity of ^223^Ra‐MOF(Fe) was much higher than that of MOF(Fe) despite of the acidic environments with different pH (Figure 2g). The pH was 4.5 has the best POD‐like enzyme activity, which is most consistent with the real TEM. Finally, we observed that the POD‐like enzyme of ^223^Ra‐MOF(Fe) increased continuously with reaction time (Figure 2h). Michaelis‐Menten curve fitting of initial reaction rates (V₀) versus H_2_O_2_ concentrations ([H_2_O_2_]) yielded a maximum reaction rate (V_max_) of 0.002904 mm·s⁻¹ and a Michaelis constant (K_m_) of 0.0 1494 mm (Figure 2i), demonstrating catalytic efficiency comparable to natural horseradish peroxidase.

This catalytic efficiency is comparable to that of natural horseradish peroxidase, suggesting ^223^Ra‐MOF(Fe) can still catalyze the conversion of H_2_O_2_ to ·OH efficiently under the acidic conditions of the tumor microenvironment. Taken together, these results confirmed that the secondary electrons emitted from ^223^Ra‐MOF(Fe) induce the production of e_aq_^−^, accelerating the realization of Fe^3+^/Fe^2+^ sustained conversion and Fe^2+^ accumulation, which subsequently enhances the Fenton reaction activity of MOF(Fe). Thus, ^223^Ra‐MOF(Fe) shows significant potential as an excellent candidate for CDT.

### 2.3 Cell Killing Efficacy of Targeting Mitochondria

**Figure 3:**
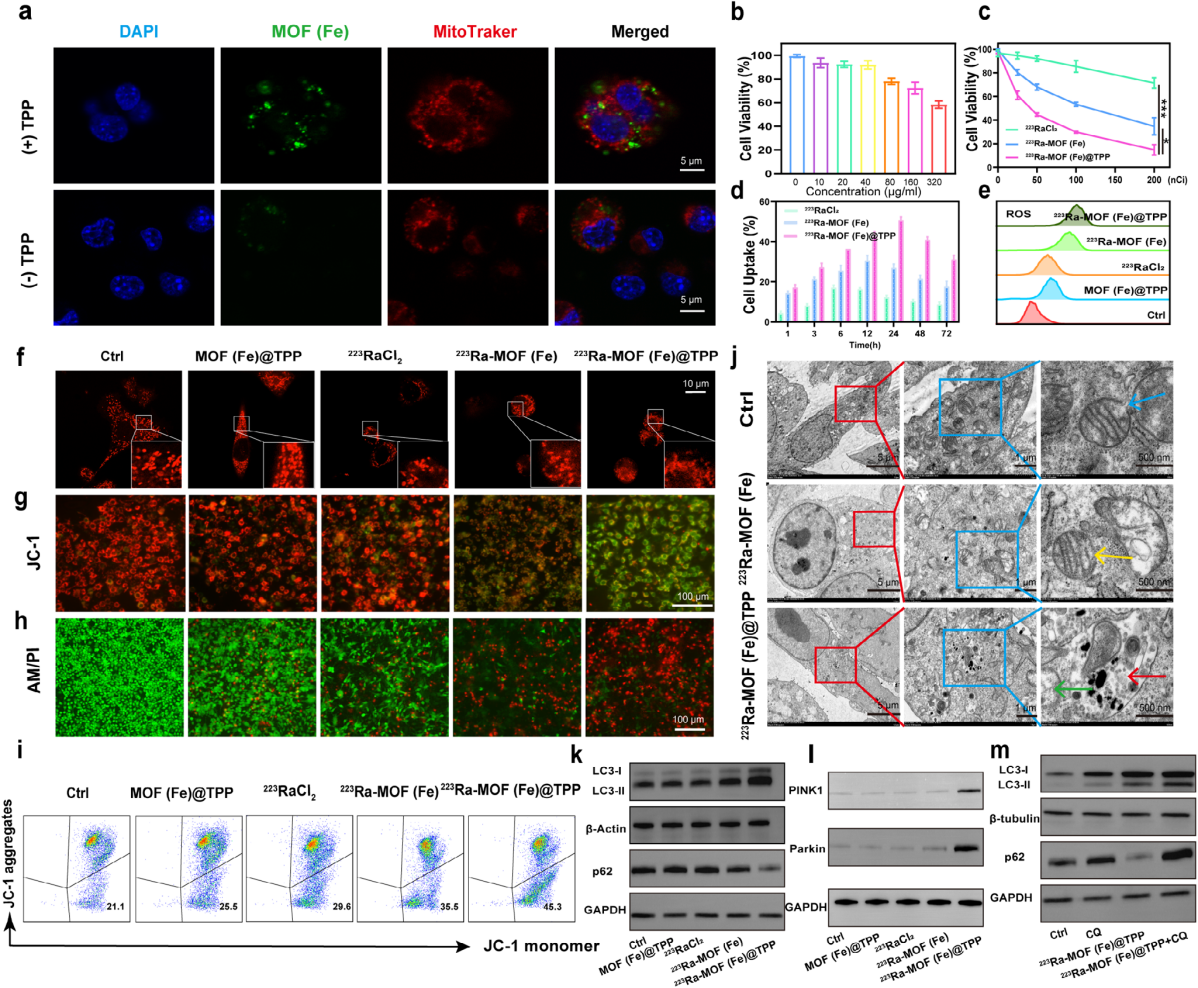
Mitochondrial dysfunction and pro‐death mitophagy were induced by ^223^Ra‐MOF(Fe)@TPP MC38 cells after being treated for 24 h. a) Colocalization of MOF(Fe) and Mito‐Tracker Red probe after 24 h co‐incubation in MC38 cells without and with TPP pretreatment. Scale bars, 5 µm. b) Cell viability of MC38 cells treated with various concentrations of MOF(Fe) for 24 h. c) Relative viability of MC38 cells treated with ^223^RaCl_2_, ^223^Ra‐MOF(Fe) and ^223^Ra‐MOF(Fe)@TPP at different doses for 24 h. d) Cell uptake assay to determine the uptake of ^223^RaCl_2_,^223^Ra‐MOF(Fe) and ^223^Ra‐MOF(Fe)@TPP by MC38 cells. e) FCM analysis of intracellular ROS. f) Confocal images of mitochondrial morphology stained with Mito‐Tracker Red. Scale bars, 10 µm. g) Immunofluorescence images of JC‐1 in MC38 cells after incubation with different formulations for 24 h. Scale bars, 100 µm. h) Representative images of MC38 cells after calcein‐AM and PI co‐staining following different treatments. Scale bars, 100 µm. i) Mitochondrial membrane potential assessment of MC38 cells under different treatments using FCM analysis. j) Representative Bio‐TEM images of mitochondrial ultrastructures in MC38 cells treated with ^223^Ra‐MOF(Fe) and ^223^Ra‐MOF(Fe)@TPP for 24 h. Normal mitochondria are seen in the Ctrl group (blue arrows), mild mitochondrial damage is seen in the ^223^Ra‐MOF(Fe) group (yellow arrows), and autophagic lysosomes (red arrows) and severely damaged mitochondria (green mitochondria) are seen in ^223^Ra‐MOF(Fe)@TPP group. Scale bars, 5 µm (left), 1 µm (middle), 500 nm(right). k) Western blot of LC3 (LC3‐I and LC3‐II) and p62 expression levels under different drug treatments (*n* = 3). l) Western blot of PINK1 and Parkin expression levels under different drug treatments (*n* = 3). m)Western blot of LC3 (LC3‐I and LC3‐II) and p62 expression levels under drug treatment and autophagy inhibitors (*n* = 3). Data are expressed as mean ± SD. ^*^ *p* < 0.05, ^**^ *p* < 0.01, ^***^ *p* < 0.001.

To evaluate the mitochondrial targeting capability of MOF(Fe)@TPP NPs in cancer cells, we observed colocalization of the green fluorescence signal from MOF(Fe)@TPP with the red fluorescence signal of the mitochondrial probe, while no such overlap was detected in the control group (**Figure** **3**a). The quantitative analysis yielded a Pearson correlation coefficient (PCC) of 0.56 ± 0.05 and a Manderson overlap coefficient (MOC) of 0.63 ± 0.06. These values indicate a significant degree of co‐localization, confirming the mitochondrial association of ^223^Ra‐MOF(Fe)@TPP. Subsequent investigation of the intracellular mechanism of this mitochondria‐targeted drug delivery system revealed critical insights into its therapeutic potential. Initial characterization through MOF(Fe) gradient concentration experiments demonstrated that its intrinsic peroxidase‐like activity significantly enhances intracellular hydroxyl radical (·OH) production,^[^ ^21^ ^]^ resulting in measurable cytotoxicity even in the absence of additional therapeutic agents (Figure 3b). Dose‐response analysis across three treatment groups, including ^223^Ra‐MOF(Fe)@TPP, ^223^Ra‐MOF(Fe), and ^223^RaCl_2_, revealed distinct cytotoxicity profiles within the 50–200 nCi range. Notably, the ^223^Ra‐MOF(Fe)@TPP group demonstrated superior therapeutic efficacy at 200 nCi, achieving a cell mortality rate of 71.4%, significantly surpassing the 34.8% and 14.9% observed in the ^223^Ra‐MOF(Fe) and ^223^RaCl_2_ groups, respectively (*p* < 0.01) (Figure 3c). Cellular uptake kinetics analysis (Figure 3d) demonstrated a characteristic pattern of initial rapid uptake followed by stabilization across all groups. The ^223^Ra‐MOF(Fe)@TPP group achieved maximal uptake at 24 h (50.7 ± 1.28%), maintaining consistently higher uptake rates compared to both ^223^RaCl_2_ (12.3%) and ^223^Ra‐MOF(Fe) (26.9%) groups throughout the 72‐h observation period. ICP‐MS analyses of intracellular iron showed that mitochondrial‐targeted cells had significantly higher iron content than non‐targeted cells (*p* < 0.01), peaking at 24 h with a slight decline at 48 h, consistent with radionuclide targeting specificity (Figure S6, Supporting Information). This sustained differential uptake profile underscores the enhanced delivery efficiency of the ^223^Ra‐MOF(Fe)@TPP nanoplatforms.

Confocal microscopy was employed to assess mitochondrial dysfunction induced by ^223^Ra‐MOF(Fe)@TPP. Control cells exhibited typical tubular mitochondrial networks with uniform distribution and intact cristae. While ^223^RaCl_2_ and ^223^Ra‐MOF(Fe) groups showed moderate mitochondrial swelling, the ^223^Ra‐MOF(Fe)@TPP group displayed severe mitochondrial fragmentation, with disrupted networks and disintegrated cristae, appearing as scattered dots or short rods (Figure 3f), quantitative data showed a significant increase in mitochondrial diameter and a significant decrease in the ^223^Ra‐MOF(Fe)@TPP group compared to the other groups (*p* < 0.05) (Figure S7, Supporting Information). Mitochondrial membrane potential (MMP) analysis further clarified the mechanism. JC‐1 immunofluorescence and flow cytometric (FCM) analysis revealed that control cells maintained normal MMP, showing predominant red fluorescence. In contrast, ^223^Ra‐MOF(Fe)@TPP treatment significantly reduced red fluorescence and increased green fluorescence (Figure 3g), indicating MMP depolarization. Cell viability assessment using calcein‐AM/PI co‐staining revealed reduced green fluorescence and increased red fluorescence in the ^223^Ra‐MOF(Fe)@TPP group (Figure 3h), indicating enhanced tumor cell apoptosis. Mitochondrial dysfunction also triggered ROS generation, as detected by DCFH‐DA probe FCM (Figure 3e; Figure S8, Supporting Information). JC‐1 FCM quantitative analysis confirmed a marked rise in low MMP cells (45.3% vs 21.1% in the control group, *p* < 0.05) (Figure 3i; Figure S9, Supporting Information), demonstrating severe functional impairment. In addition, apoptotic FCM analysis was supported by a significantly higher apoptosis rate of 78.7% in the ^223^Ra‐MOF(Fe)@TPP group compared to other groups (10.5%, 25.6%, 31.6%, 47%) (*p* < 0.01) (Figure S10, Supporting Information), the immunofluorescence results of ki67 were consistent with these results (Figure S11, Supporting Information). Collectively, these findings demonstrated that ^223^Ra‐MOF(Fe)@TPP effectively induces mitochondrial dysfunction, leading to substantial tumor cell death.

Mitochondrial integrity disruption and ROS overproduction typically trigger mitochondrial autophagy, a process that eliminates damaged mitochondria via lysosomal degradation.^[^ ^22^ ^]^ Bio‐TEM images revealed that cells treated with ^223^Ra‐MOF(Fe) exhibited only mild mitochondrial swelling, while ^223^Ra‐MOF(Fe)@TPP treatment led to the formation of autophagic lysosomes, characterized by damaged mitochondria enclosed within monolayer membrane structures (Figure 3k). Furthermore, to evaluate the potential of ^223^Ra‐MOF(Fe)@TPP to induce autophagy, light chain protein 3 (LC3) expression, and p62 protein were analyzed using immunoprecipitation. Treatment with ^223^Ra‐MOF(Fe)@TPP significantly enhanced the conversion of LC3‐I to lipidated LC3‐II and concurrently reduced the expression level of the autophagy substrate p62 (Figure 3k; Figure S12, Supporting Information), indicating the induction of autophagy. We first confirmed ^223^Ra‐MOF(Fe)@TPP‐induced autophagy through increased LC3‐II accumulation and p62 degradation. Subsequent experiments revealed upregulation of mitophagy markers (PINK1 and Parkin) (Figure 3l; Figure S12, Supporting Information), indicating mitochondrial autophagy activation. Crucially, treatment with autophagy inhibitors (chloroquine) simultaneously suppressed both the initial autophagy flux (LC3‐II/p62), establishing autophagy as the essential upstream driver of mitophagy in this system (Figure 3m; Figure S13, Supporting Information).

These findings suggested that mitochondria‐targeted drugs exert therapeutic effects by activating autophagy‐driven mitophagy and mitochondria‐dependent cell death pathways. These results demonstrated that mitochondria‐targeting strategies can achieve selective cytotoxicity through a dual mechanism that includes ROS‐mediated mitochondrial damage and autophagy activation.

### 2.4 Induction of Mitochondrial Autophagy for ICD Death

**Figure 4:**
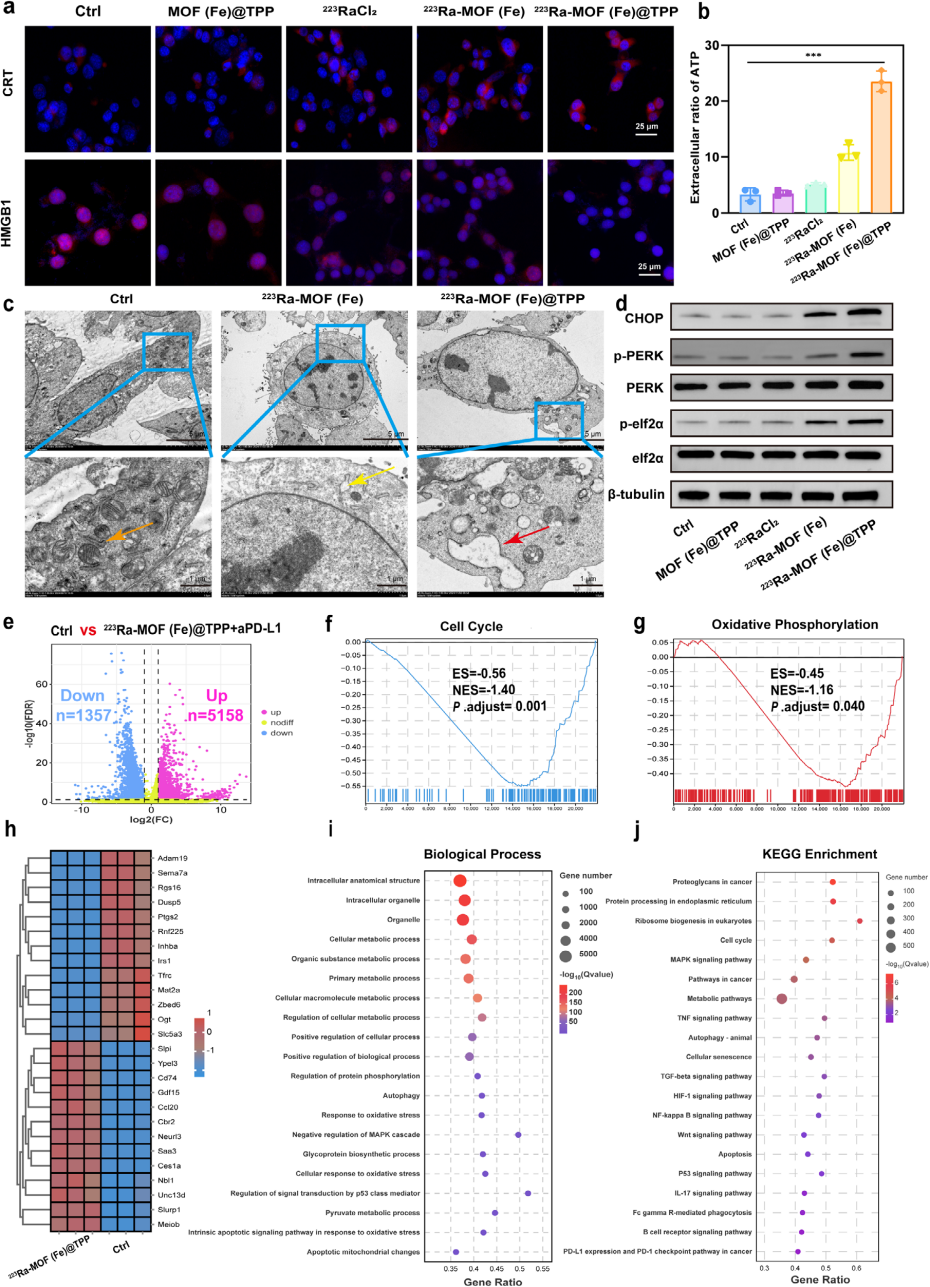
^223^Ra‐MOF(Fe)@TPP induces ICD. a) Immunofluorescence images of CRT and HMGB1 in MC38 cells after incubation with ^223^Ra‐MOF(Fe)@TPP treatment for 24 h. Scale bars, 25 µm. b) Quantification analysis of ATP in the supernatant of MC38 cells after treated for 24 h (*n* = 3; mean ± SD). c) Bio‐TEM visualization of the ER change of untreated, after treatment with ^223^Ra‐MOF(Fe) and ^223^Ra‐MOF(Fe)@TPP for 24 h. Normal endoplasmic reticulum is seen in the Ctrl group (orange arrows), mild endoplasmic reticulum damage is seen in the ^223^Ra‐MOF(Fe) group (yellow arrows), and severe endoplasmic reticulum swelling is seen in the ^223^Ra‐MOF(Fe)@TPP group (red arrows). Scale bars, 5 µm(Top), 1 µm (Bottom). d) Representative western blot of indicated proteins in MC38 cells after different treatments. e) The volcano plot of DEGs in ^223^Ra‐MOF(Fe)@TPP group versus the control group. f) GSEA revealing negative enrichment of DEGs in cell cycle processes. g) GSEA revealing negative enrichment of DEGs in oxidative phosphorylation processes. h) Differential gene expression heat maps of identified genes involved in mitophagy, ER stress, regulated cell death, and immune and inflammatory pathways. i) GO categorization of biological process for DEGs induced by ^223^Ra‐MOF(Fe)@TPP treatment. j) KEGG enrichment analysis for DEGs induced by ^223^Ra‐MOF(Fe)@TPP treatment. Data are expressed as mean ± SD. ^*^ *p* < 0.05, ^**^ *p* < 0.01, ^***^ *p* < 0.001.

Mitochondrial autophagy, a critical biological process, initiates the release of immunogenic ATP from dying cells.^[^ ^23^ ^]^ To validate this mechanism, we analyzed two additional ICD markers, calreticulin (CRT) and high mobility group protein 1 (HMGB1).^[^ ^24^ ^]^ Fluorescence imaging demonstrated that CRT expression on the cell surface was significantly higher in the ^223^Ra‐MOF(Fe)@TPP group compared to other groups (**Figure** **4**a). HMGB1 immunofluorescence revealed its translocation and release in the ^223^Ra‐MOF(Fe)@TPP group, with fluorescence shifting from the nucleus to the cytoplasm and extracellular space, accompanied by a marked reduction in nuclear fluorescence intensity. We further measured ATP concentrations in cell supernatants, revealing that extracellular ATP secretion in the ^223^Ra‐MOF(Fe)@TPP‐treated group increased more than 2‐fold compared to the ^223^RaCl_2_ and ^223^Ra‐MOF(Fe) groups (Figure 4b). These findings confirmed that ^223^Ra‐MOF(Fe)@TPP effectively induces tumor cell immunogenic death. To elucidate the molecular mechanisms driving CRT exposure, we performed comprehensive mechanistic investigations. Bio‐TEM images unveiled distinct endoplasmic reticulum (ER) swelling in cells treated with ^223^Ra‐MOF(Fe)@TPP (Figure 4c), a hallmark of ER homeostasis disruption. Western blot analysis further showed significantly elevated CHOP and p‐eIF2α/eIF2α and p‐PERK/PERK ratios in ^223^Ra‐MOF(Fe)@TPP treatment group compared to the control group (Figure 4d; Figure S14, Supporting Information). In experiments involving the endoplasmic reticulum stress inhibitor 4‐Phenylbutyric acid (4‐PBA), we observed significant changes in the expression of proteins that are key markers of endoplasmic reticulum stress. CHOP, p‐PERK, and p‐eIF2α levels were higher in the group treated with the mitochondria‐targeting drug ^223^Ra‐MOF(Fe)@TPP alone. Treatment with 4‐PBA alone effectively reduced the expression levels of the phosphorylated protein markers p‐PERK and p‐eIF2α, as well as CHOP. Most critically, the treatment group that received a combination of ^223^Ra‐MOF(Fe)@TPP and 4‐PBA showed a significant reduction in CHOP, p‐PERK, and p‐eIF2α protein levels compared with the group that received ^223^Ra‐MOF(Fe)@TPP alone, suggesting that 4‐PBA effectively inhibits the activation of the ER stress signaling pathway induced by ^223^Ra‐MOF(Fe)@TPP. Notably, total PERK and total eIF2α protein expression remained relatively stable without significant changes in any of the treatment groups (Figures S15 and S16, Supporting Information). These results suggested that mitochondrial dysfunction triggers ER stress, activating the PERK/eIF2α phosphorylation pathway and driving CRT transmembrane transport.

RNA sequencing (RNA‐seq) was performed to assess the impact of ^223^Ra‐MOF(Fe)@TPP on gene expression in MC38 cells. A volcano plot identified 6515 differentially expressed genes (DEGs), with 5158 upregulated and 1357 downregulated in the ^223^Ra‐MOF(Fe)@TPP group versus the control group (Figure 4e). Gene set enrichment analysis (GSEA) revealed negative correlations with the cell cycle (Figure 4f) and oxidative phosphorylation Figure 4g). A gene expression heatmap confirmed the upregulation of immune‐related genes (Ccl20, Cd74, Gdf15) in the experimental group, linked to PD‐L1/PD‐1 checkpoint pathway activation. This supports the hypothesis that CRT exposure enhances immunogenic signaling via the PERK‐eIF2α phosphorylation cascade. Upregulated pro‐inflammatory factors (Ccl20, Saa3) and downregulated mitochondrial metabolic genes (Slc5a3, Mat2a) (Figure 4h) further emphasized the pivotal role of mitochondria in immunogenic death. Gene ontology (GO) enrichment analysis highlighted significant DEG involvement in cellular metabolic processes, autophagy, oxidative stress response, mitochondrial apoptosis signaling, and negative regulation of the MAPK cascade (Figure 4i). Additionally, the enrichment of pyruvate metabolism and intrinsic apoptotic signaling pathways underscores the central role of mitochondrial oxidative damage in programmed cell death. KEGG pathway analysis revealed significant DEG enrichment in autophagy, apoptosis, P53 signaling, and IL‐17 signaling pathways (Figure 4j; Figure S17, Supporting Information), indicating functional coupling between damage and ER stress. In summary, targeted mitochondrial damage triggers ICD by activating the PERK‐eIF2α pathway through dual mechanisms: pro‐death autophagy and ER stress, thereby enhancing anti‐tumor immunity.

### 2.5 Targeted Delivery Evaluation for Enhanced Tumor Retention, Immunomodulation, and Biodistribution

For high LET radiotherapeutic agents like the α emitter, ^223^Ra, therapeutic efficacy is critically dependent on achieving local drug concentrations within tumors. Intravenous drug delivery often fails to reach effective levels in tumors because their abnormal blood vessels hinder proper drug accumulation.^[^ ^25^, ^26^ ^]^ In recent years, interventional radiology‐guided intratumoral injection has gained significant attention for its ability to bypass vascular barriers and enable precise drug localization.^[^ ^27^ ^]^ This approach theoretically avoids first‐pass metabolism and establishes a localized high‐concentration drug platform, which is particularly advantageous for TAT.

**Figure 5:**
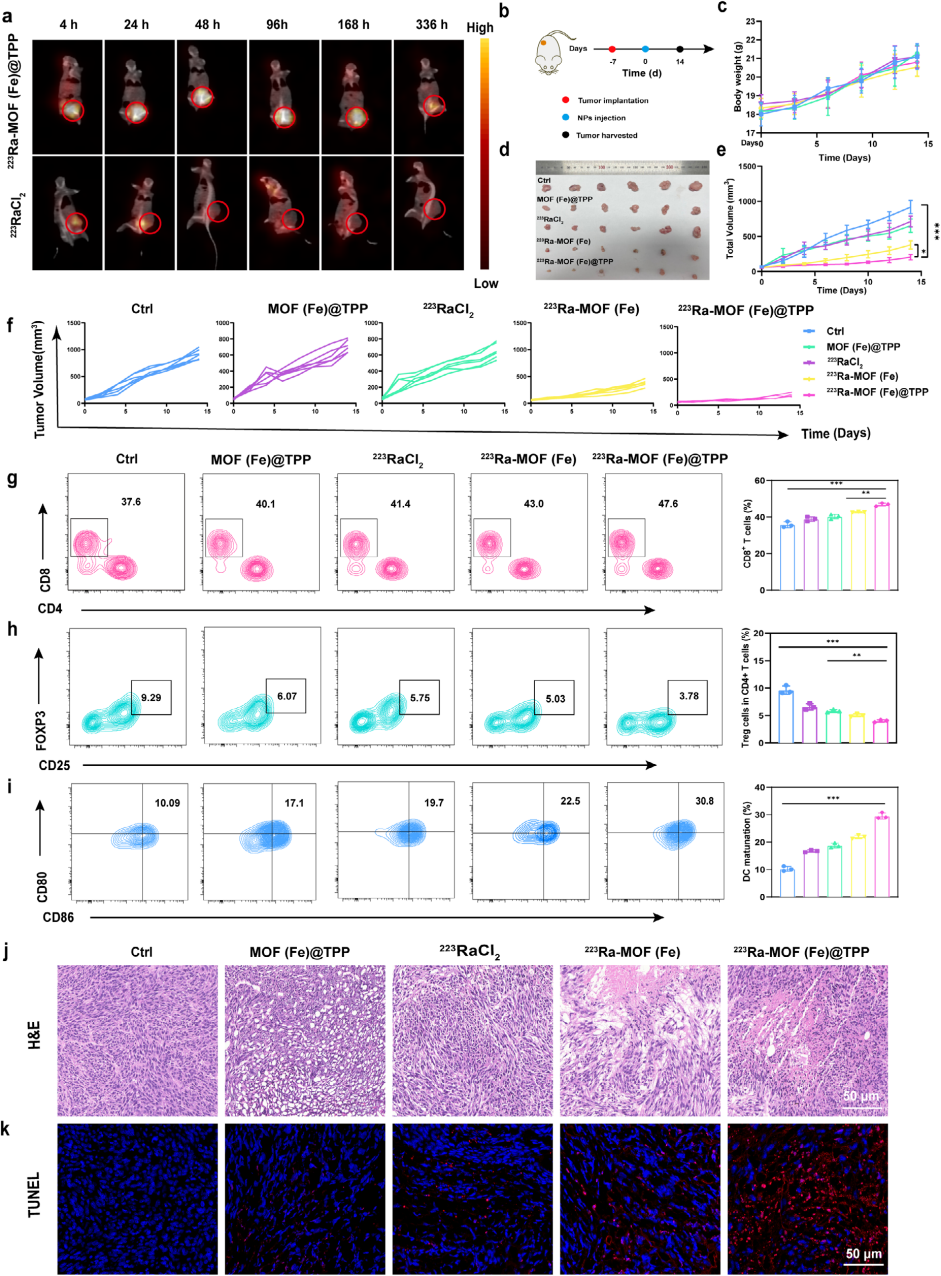
In vivo evaluation of anti‐tumor effects. a) SPECT/CT images of MC38 tumor‐bearing mice following intratumoral injection of free ^223^RaCl_2_ and ^223^Ra‐MOF(Fe)@TPP. b) Schematic representation of the experimental design for in vivo antitumor evaluations. c) Body weight changes after different formulations treatment (*n* = 6). d) Photographs of tumors and e) corresponding tumor weights in each group of mice on the 14th day after different treatments (*n* = 6). f) Quantitative analysis of tumor growth inhibition rates in each group of mice on the 14th day after different treatments (*n* = 6). g) FCM analysis and quantification of mature CD8⁺ T cells in tumors of mice after different treatments (*n* = 3). h) FCM analysis and quantification of Tregs in tumors of mice after different treatments. (*n* = 3). i) FCM analysis and quantification of DC cell (CD80^+^ CD86^+^) in tumors of mice after different treatments. (*n* = 3). j) TUNEL staining images of primary tumors k) and H&E staining images of tumors in mice after various treatments. Scale bars, 50 µm. Data are expressed as mean ± SD. ^*^ *p* < 0.05, ^**^ *p* < 0.01, ^***^ *p* < 0.001.

We compared the efficacy and in vivo biodistribution of two delivery methods (intravenous and intratumoral injection) in tumor‐bearing mice using SPECT/CT imaging to assess the potential of targeted α‐particle delivery. Results revealed that intratumorally administered ^223^Ra‐MOF(Fe)@TPP exhibited significant tumor retention with minimal non‐target organ uptake, whereas intravenously delivered ^223^Ra‐MOF(Fe)@TPP primarily accumulated in the reticuloendothelial system, with only modest tumor uptake (Figure S18, Supporting Information). Consequently, the intratumoral injection was selected for further experimentation. Imaging of mice treated with ^223^RaCl_2_ (500 nCi) and ^223^Ra‐MOF(Fe)@TPP (500 nCi) demonstrated rapid diffusion of ^223^RaCl_2_ from the tumor site post‐injection. In contrast, the ^223^Ra‐MOF(Fe)@TPP group showed sustained drug retention in the tumor region over 336 h (**Figure** **5**a), confirming its superior tumor targeting and stability. Subsequent γ‐counter analysis of radioactivity distribution in tumor and vital organs at 4 h, 8 h, and 24 h (Figures S19 and S20, Supporting Information) revealed significant bone accumulation of ^223^RaCl_2_ as early as 4 h post‐injection. In contrast, ^223^Ra‐MOF(Fe)@TPP primarily localized to the tumor, with minimal uptake in non‐target organs such as bone, liver, and spleen, consistent with imaging findings.

To assess the direct antitumor efficacy of mitochondria‐targeted therapy, a subcutaneous MC38 tumor model was established (Figure 5b). Throughout the treatment period, mouse body weights remained stable (Figure 5c), and no significant differences were observed in renal function, routine blood parameters, or histopathological assessments of major organs (heart, liver, spleen, lungs, and kidneys), indicating negligible systemic toxicity (Figures S21–S23, Supporting Information) Physical tumor maps confirmed the smallest tumor volume in the ^223^Ra‐MOF(Fe)@TPP group (Figure 5d), aligning with its pro‐apoptotic effects observed in vitro. Tumor growth was markedly suppressed in the ^223^Ra‐MOF(Fe)@TPP group, with mean tumor volumes at treatment endpoint measuring 918.7 mm^3^ (Control), 712.2 mm^3^ (MOF(Fe)@TPP), 652.4 mm^3^ (^223^RaCl_2_), 377.5 mm^3^ (^223^Ra‐MOF(Fe)), and 202.8 mm^3^ (^223^Ra‐MOF(Fe)@TPP) (Figures 5e,f).

FCM analysis revealed that ^223^Ra‐MOF(Fe)@TPP significantly increased intratumoral CD8⁺ T cell infiltration (47.6% vs 37.6% in the control group) (Figure 5g; Figures S24 and S25, Supporting Information) and reduced immunosuppressive regulatory T cell (Tregs) populations (3.78% vs 9.29%) (Figure 5h). Additionally, mature dendritic cell (DC) proportions in tumors reached 30.8%, threefold higher than the control group (Figure 5i), indicating potent activation of adaptive immunity. Histological analysis (H&E staining) revealed extensive necrotic areas in ^223^Ra‐MOF(Fe)@TPP‐treated tumors, with marked nuclear condensation and cytoplasmic dissolution (Figure 5j). TUNEL fluorescence imaging further confirmed significantly higher apoptotic cell fluorescence intensity in this group compared to others (Figure 5k). In summary, ^223^Ra‐MOF(Fe)@TPP demonstrated potent localized antitumor activity by precisely targeting mitochondria, enhancing tumor‐specific drug accumulation, and activating the antitumor immune microenvironment, while effectively reducing bone toxicity risks inherent to conventional TAT.

### 2.6 Distant Effects and Immunomodulation of Combined Immunotherapy

**Figure 6:**
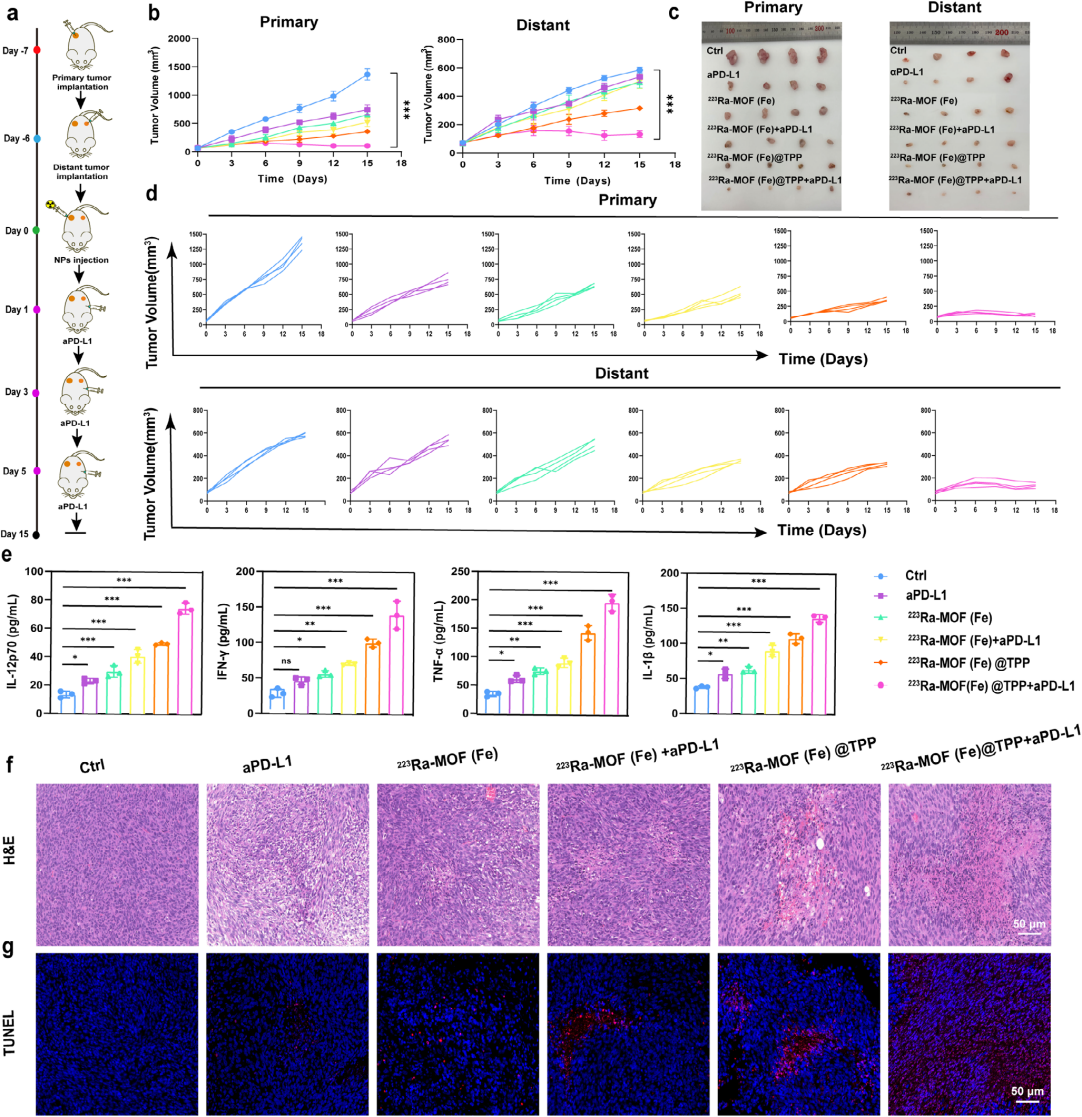
^223^Ra‐MOF(Fe)@TPP combined with aPD‐L1 enhances abscopal effects in vivo. a) Schematic illustration of constructing bilateral MC38 tumor models. b) Average growth curves of primary and distant tumors in MC38 mice after indicated treatments (*n* = 4). c) Photographs of primary and distant tumors. d) Individual growth curves of primary and distant tumors in MC38 mice after indicated treatments (*n* = 4). e) IL‐12p70, IFN‐γ, TNF‐α and IL‐1β levels in the serum of MC38 bilateral tumor model mice after various treatments (*n* = 3). f) Immunofluorescence images of TUNEL in distant tumors after different treatments. Scale bars, 50 µm. g) H&E staining images of distant tumors after different treatments. Scale bars, 50 µm. Data are expressed as mean ± SD. ^*^ *p* < 0.05, ^**^ *p* < 0.01, ^***^ *p* < 0.001.

Previous studies have demonstrated that the immunosuppressive tumor microenvironment significantly restricts the abscopal effects of radiotherapy, whereas a remodeled tumor microenvironment can enhance these effects by promoting the infiltration of immune cells, including CD8^+^ T cells, regulatory T cells, and M1‐type macrophages.^[^ ^28^, ^29^ ^]^ To explore this further, we evaluated the combined efficacy of ^223^Ra‐MOF(Fe)@TPP + anti‐PD‐L1 therapy (abbreviated as aPD‐L1) in inhibiting distant tumor growth using an MC38 bilateral tumor model (**Figure** **6**a). Tumor growth curves revealed that ^223^Ra‐MOF(Fe)@TPP + aPD‐L1 not only suppressed primary tumor growth but also significantly inhibited distant tumor progression (Figure 6b–d). The combined treatment group exhibited a marked reduction in tumor volume compared to the control group, with statistical significance (*p* < 0.01) observed at all measured time points. No significant changes in body weight were observed across treatment groups (Figure S26, Supporting Information), indicating the safety of ^223^Ra‐MOF(Fe)@TPP + aPD‐L1. In contrast, monotherapy with aPD‐L1 or ^223^Ra‐MOF(Fe) only moderately restrained primary tumor growth, with minimal impact on distant tumors (Figure 6b–d). Specifically, aPD‐L1 therapy showed a transient delay in primary tumor growth during the initial phase of treatment, but this effect diminished over time, and no significant inhibition of distant tumors was observed. Similarly, ^223^Ra‐MOF(Fe) monotherapy demonstrated localized antitumor activity at the primary site, likely due to the targeted delivery of radionuclides, but failed to exert a systemic effect on distant lesions. The enhanced efficacy of the combination therapy can be attributed to the synergistic interaction between the localized radiation‐induced ICD mediated by ^223^Ra‐MOF(Fe)@TPP and the systemic immune activation facilitated by aPD‐L1 therapy. The radiation component likely promoted the release of tumor‐associated antigens, thereby enhancing the recruitment and activation of cytotoxic T cells. Concurrently, aPD‐L1 therapy prevented T cell exhaustion and sustained an active immune response against both primary and distant tumors. These findings underscore the potential of combining targeted radionuclide therapy with immune checkpoint blockade to achieve both local and systemic tumor control, offering a promising strategy for treating metastatic cancers.

At the study endpoint, serum cytokine levels were quantified to assess systemic immune activation, which is critical for understanding the therapeutic mechanisms underlying the observed antitumor effects.^[^ ^30^ ^]^ ELISA results showed that the ^223^Ra‐MOF(Fe)@TPP + aPD‐L1 group exhibited a 5.6‐fold increase in IL‐12p70, a 3.6‐fold increase in IL‐1β, a 4.8‐fold increase in IFN‐γ, and a 5.5‐fold increase in TNF‐α levels compared to the untreated group (Figure 6e). These cytokines were key mediators of antitumor immunity, and their marked upregulation suggested an intense systemic immune response. Specifically, the significant elevation of IL‐12p70 indicated strong Th1 immune activation, which is essential for promoting cellular immunity and cytotoxic T‐cell responses. The sustained high levels of IFN‐γ further supported enhanced cytotoxic T‐cell activity, which is critical for targeting both primary and distant tumors. Additionally, the increased levels of IL‐1β and TNF‐α highlighted the activation of pro‐inflammatory pathways, contributing to the destruction of tumor cells and the recruitment of immune cells to the tumor mircroenvironment. In contrast, monotherapy with either aPD‐L1 or ^223^Ra‐MOF(Fe) alone only modestly elevated cytokine concentrations. These results suggest that while monotherapy can partially activate the immune system, the combination therapy synergistically amplifies immune activation, leading to a more potent and sustained antitumor response.

The inhibitory effect on distant metastasis was further corroborated by histological analyses, including TUNEL and H&E staining. In the ^223^Ra‐MOF(Fe)@TPP + aPD‐L1 group, H&E staining confirmed significant tumor tissue damage, including necrosis and immune cell infiltration in metastatic sites. In contrast, H&E staining revealed limited tissue damage and immune cell infiltration in distant tumors treated with monotherapy (Figure 6f). TUNEL staining further revealed extensive tumor cell apoptosis in distant tumors. These findings collectively demonstrate that the combination of ^223^Ra‐MOF(Fe)@TPP and aPD‐L1 not only enhances local tumor control but also induces a systemic immune response capable of targeting metastatic lesions (Figure 6g).

**Figure 7:**
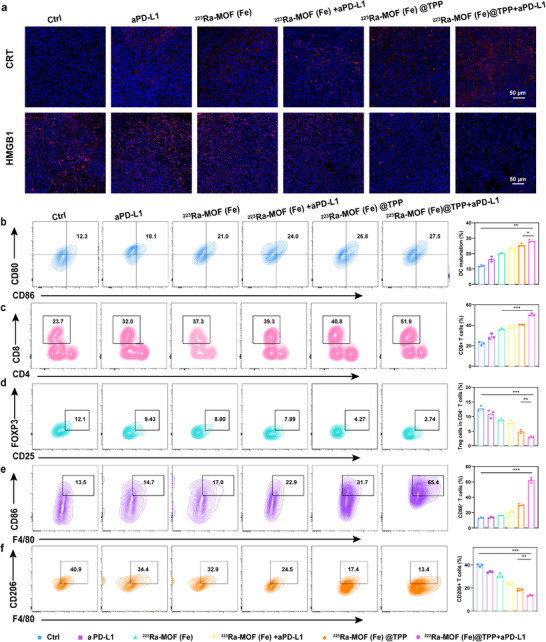
^223^Ra‐MOF(Fe)@TPP combined with aPD‐L1 therapy enhances immune response in vivo. a) Immunofluorescence images of CRT and HMGB1 in distant tumors after different treatments. Scale bars,50 µm. b) FCM analysis of tumor DC maturation with quantitative analysis. c) FCM analysis of tumor CD8^+^ T cell with quantitative analysis. d) FCM analysis of tumor Tregs cell with the quantitative analysis. e) FCM analysis of tumor M1 type macrophages with the quantitative analysis. f) FCM analysis of tumor M2 type macrophages with quantitative analysis. Data are expressed as mean ± SD. ^*^ *p* < 0.05, ^**^ *p* < 0.01, ^***^ *p* < 0.001.

To further investigate the modulation of the tumor immune microenvironment, we assessed ICD markers, which are critical for initiating antitumor immune responses. Distant tumors in the ^223^Ra‐MOF(Fe)@TPP group exhibited significantly increased CRT membrane exposure and elevated extracellular release of high mobility group protein HMGB1 (**Figure** **7**a). CRT, a well‐known “eat‐me” signal, facilitates the phagocytosis of dying tumor cells by antigen‐presenting cells (APCs), while HMGB1 acts as a damage‐associated molecular pattern (DAMP) that promotes DC activation and antigen presentation (Figure 7a). These findings confirmed the induction of immunogenic apoptosis by mitochondria‐targeted therapy, which is a key mechanism for eliciting antitumor immunity.

Mechanistic studies revealed DC maturation in the ^223^Ra‐MOF(Fe)@TPP group (Figure 7b), this indicated improved antigen presentation and subsequent T cell priming, which are essential for the initiation of adaptive immune responses. Additionally, the combination therapy group showed a significant increase in CD8^+^ T cell infiltration (2.19‐fold higher than the control group) and a marked reduction in Tregs (2.74% vs 12.1% in the control group) (Figure 7c,d), which are known to suppress immune responses, further highlights the ability of aPD‐L1 therapy to amplify antitumor immunity by alleviating immunosuppression. Furthermore, tumor‐associated macrophage (TAM) polarization was analyzed to evaluate the impact of the therapy on the innate immune compartment. The proportion of tumor‐suppressive M1 type macrophages, which exhibit antitumor properties, increased significantly in the ^223^Ra‐MOF(Fe)@TPP and combination groups (31.7% and 65.4%, respectively, versus 13.5% in the control group). Conversely, the proportion of tumor‐promoting M2 type macrophages, which are associated with immunosuppression and tumor progression, decreased (17.4% and 13.4%, respectively, versus 40.9% in the control group) (Figure 7e,f). This shift in macrophage polarization toward an M1 type phenotype further supports the immunostimulatory effects of the therapy. These results emphasized the ability of ^223^Ra‐MOF(Fe)@TPP + aPD‐L1 to positively reshape the tumor immune landscape by inducing ICD, enhancing DC maturation, promoting CD8^+^ T cell infiltration, reducing Tregs, and reprogramming TAMs toward an antitumor phenotype. In summary, ^223^Ra‐MOF(Fe)@TPP‐based mitochondria‐targeted therapy induces potent ICD, eliciting a robust adaptive antitumor immune response. This effect is synergistically enhanced by aPD‐L1 therapy, which mitigates immunosuppression and amplifies systemic immunity, presenting a promising approach for metastatic cancer treatment. The combination of these therapies not only targets the primary tumor but also generates a systemic immune response that may prevent metastatic spread and recurrence, offering a potential breakthrough in cancer immunotherapy.

### 2.7 Long‐Term Immune Memory Response Induced by ^223^Ra‐MOF(Fe)@TPP Combined with aPD‐L1 Inhibits Lung Metastasis

**Figure 8:**
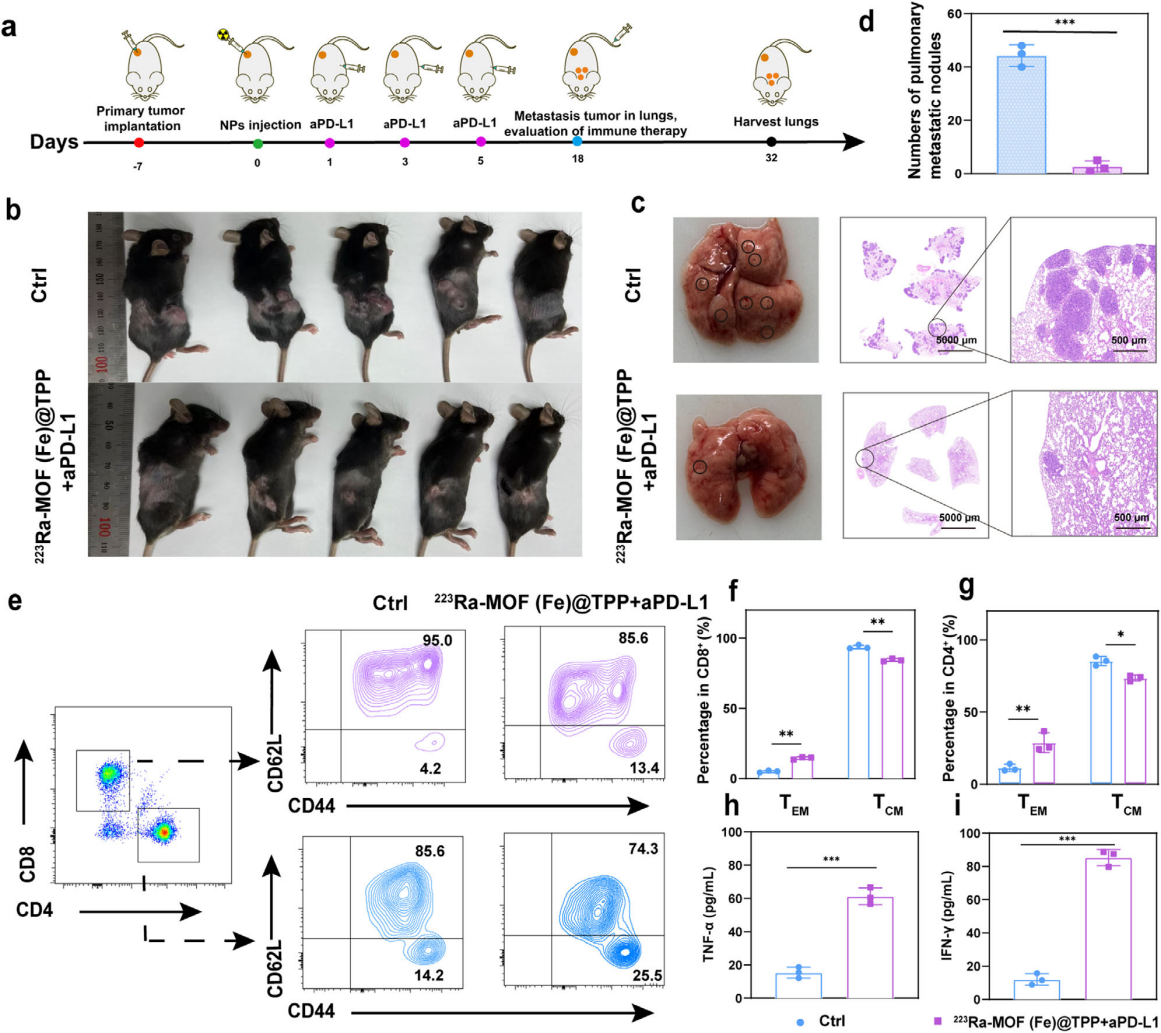
The immune memory response triggered by the combination of ^223^Ra‐MOF(Fe)@TPP and aPD‐L1 therapy enhances the inhibition of lung metastasis. a) Schematic diagram of experimental design to evaluate the immune memory response triggered by ^223^Ra‐MOF(Fe)@TPP enhanced RT and aPD‐L1 therapy enhanced the inhibition of lung metastasis. b) Representative tumor photographs of the primary tumor from each group after varied treatments as indicated. c) Representative photographs of lung tissue images from each group after treatments (the black circles indicate the metastatic nodules) and H&E staining at the end of treatment in each group (*n* = 5). Scale bars, 5000 µm, 500 µm. d) Corresponding quantified pulmonary metastatic nodules. e) Representative FCM analysis and relative quantification of central memory T cell (T_CM_, CD62L^+^CD44^+^) and effector memory T cell (T_EM_, CD62L^−^CD44^+^) subset from CD8^+^ f) and CD4^+^ g) T cells in the spleen. Cytokine levels of TNF‐α h) and IFN‐γ i) in the serum after tumor rechallenging. Data are expressed as mean ± SD. ^*^ *p* < 0.05, ^**^ *p* < 0.01, ^***^ *p* < 0.001.

As a hallmark of adaptive immunity, immune memory establishes a durable defense mechanism, playing a pivotal role in protecting against pathogen reinfection and tumor recurrence.^[^ ^31^, ^32^ ^]^ To investigate the immune memory effects elicited by ^223^Ra‐MOF(Fe)@TPP + aPD‐L1, we assessed its efficacy in an MC38 tumor lung metastasis model. Following the eradication of primary MC38 tumors by combination therapy, mice were rechallenged with intravenous injection of MC38 tumor cells to induce lung metastasis (**Figure** **8**a). Age and sex‐matched control mice inoculated with the same tumor cell number served as comparators. Results revealed rapid subcutaneous tumor growth (Figure 8b) and aggressive lung metastasis (characterized by nuclear heterogeneity and interstitial infiltration) in the control group (Figure 8c). In contrast, the ^223^Ra‐MOF(Fe)@TPP + aPD‐L1 group exhibited very few lung metastatic foci (Figure 8c,d). There was no difference in body weight between the two groups, suggesting that the treatment was safe (Figure S27, Supporting Information).

To elucidate the molecular mechanisms underlying immune memory formation, spleen tissues were collected 14 days post‐secondary tumor inoculation for FCM analysis, with a focus on memory T cell subsets. Antigen‐specific memory T cells are primarily categorized into central memory T cells (T_CM_) (CD4^+^, CD8⁺, CD62L^+^, CD44^+^) and effector memory T cells (T_EM_) (CD4^+^, CD8^+^, CD62L^−^, CD44^+^), rapidly recognize tumor antigens and execute immediate immune defense through cytokine secretion, such as TNF‐α and IFN‐γ. Notably, CD8^+^ T and CD4^+^ T cell‐derived T_CM_ in long‐term survivors treated with ^223^Ra‐MOF(Fe)@TPP + aPD‐L1 showed a significant shift toward a T_EM_ phenotype compared to untreated cells (Figure 8e–g).

These findings collectively demonstrate that the combined ^223^Ra‐MOF(Fe)@TPP+ aPD‐L1 therapeutic regimen successfully establishes an effective antitumor immune memory barrier by reprogramming the distribution of memory T cell subsets and enhancing systemic cytokine responses. This provides a solid immunological foundation for suppressing tumor recurrence and metastasis. Concurrently, serum levels of key antitumor cytokines, including TNF‐α and IFN‐γ, were markedly elevated in the combination therapy group (Figure 8h,i). The increased levels of TNF‐α and IFN‐γ in the serum suggest a heightened state of immune activation, which is essential for the rapid and effective response to tumor rechallenge. Additionally, the reduction in lung metastatic foci in the treated group underscores the potential of the therapeutic approach in preventing distant metastasis.

Moreover, the shift from T_CM_ to T_EM_ phenotype indicates a more active and responsive immune state, capable of mounting a swift and potent defense against tumor cells. This phenotypic shift is likely driven by the unique properties of ^223^Ra‐MOF(Fe)@TPP, which may enhance antigen presentation and T cell priming, in synergy with the immune checkpoint blockade provided by aPD‐L1. Together, these mechanisms contribute to the establishment of a durable and effective immune memory, offering a promising strategy for long‐term cancer control and prevention of distant metastasis.

### 2.8 Evaluation of Combined Low‐Dose Radiotherapy with aPD‐L1 Therapy for Anti‐Tumor Efficacy

**Figure 9:**
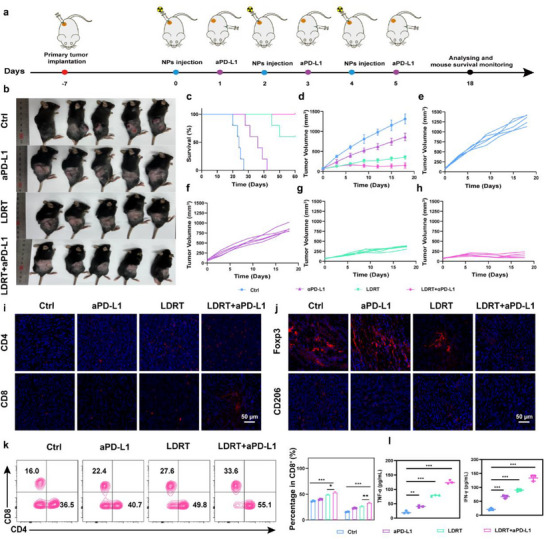
Evaluation of the Antitumor Efficacy of Low‐Dose Radiation Therapy Combined with aPD‐L1 therapy. a) Schematic illustration of constructing LDRT MC38 tumor models. b) Representative tumor photographs tumor after treatments. c) Survival curves in MC38 tumor‐bearing mice after indicated treatments (*n* = 5). d) The tumor growth curves and e–h) corresponding individual growth curves of MC38 tumor‐bearing mice in each group (*n* = 5). i) Immunofluorescence images of CD4⁺ and CD8⁺ T cells after different treatments. Scale bars, 50 µm. j) Immunofluorescence images of FOXP3^+^ and CD206^+^ M2 type macrophage in tumors after different treatments. Scale bars, 50 µm. k) Representative FCM analysis of CD8⁺ and CD4⁺ T cells in the spleen with quantitative analysis. l) Cytokine levels of TNF‐α and IFN‐γ in the serum after treatment. Data are expressed as mean ± SD. ^*^ *p* < 0.05, ^**^ *p* < 0.01, ^***^ *p* < 0.001.

The scientific and clinical significance of the study of multiple low‐dose radiation(LDRT) therapy is evident.^[^ ^33^, ^34^ ^]^ The reduction of normal tissue damage, overcoming tumor heterogeneity, and reduction of toxic side effects are the most salient advantages of this therapy.^[^ ^35^ ^]^ Furthermore, the strategy of employing LDRT has been shown to accumulate immune stimulatory signals, thereby continuously “initiating” and “strengthening” the anti‐tumor immune response. This effect is analogous to the outcome of multiple immunizations. Additionally, the strategy's superior safety profile enables its combination with drugs such as aPD‐L1. This is of particular significance for high LET radiation (α‐nuclides). The selection of a split irradiation regimen, characterized by low physical doses, constitutes a pivotal approach to achieving a balance between the potent antitumor activity of α‐nuclides and the potential for toxic side effects. Based on this, in order to elucidate the synergistic antitumor effects of LDRT combined with immune checkpoint blockade, we established a subcutaneous tumor model in female C57 mice (**Figure** **9**a). Therapeutic outcomes demonstrated marked tumor regression in the LDRT + aPD‐L1 group, exhibiting superior efficacy compared to monotherapy regimens (Figure 9b). Survival analysis revealed prolonged median survival exceeding 60 days in the combination therapy group (Figure 9c). The tumor growth curves further corroborated the therapeutic efficacy observed in the LDRT+aPD‐L1 group. Overall tumor growth in the combination therapy group showed a significant suppression compared to monotherapy groups (LDRT alone and aPD‐L1 alone) and the control group. Specifically, tumors in the LDRT+aPD‐L1 group exhibited a delayed onset of growth and a markedly reduced growth rate, and some tumors even approached regression over time. (Figure 9d). In contrast, tumors in the monotherapy groups displayed partial growth inhibition, while the control group exhibited rapid and unchecked tumor progression. Individual tumor growth curves highlighted the heterogeneity in response within each treatment group (Figure 9e–h). These findings underscore the synergistic effect of combining LDRT with aPD‐L1, which not only enhances overall tumor control but also improves the consistency of therapeutic outcomes across individual subjects. Immunofluorescence quantification showed enhanced CD4^+^ and CD8^+^ T cell infiltration within tumor microenvironment following combination treatment, with fluorescence intensities surpassing those observed in LDRT alone, aPD‐L1 monotherapy, and the control group groups (Figure 9i). Notably, the combined regimen substantially reduced immunosuppressive cell populations, demonstrating decreased Treg frequencies and diminished CD206^+^ M2 type macrophage polarization, indicating effective modulation of tumor‐associated immunosuppression (Figure 9j).

FCM analysis of splenic lymphocytes revealed augmented cytotoxic T cell activation, with CD8^+^ T cell proportions increasing to 33.6% (vs 16.0% in the control group, *p* < 0.001) and CD4^+^ T cell populations rising to 55.1% (vs 36.5% in the control group) (Figure 9k). Cytokine profiling confirmed enhanced Th‐type immune activation, showing elevated TNF and IFN‐γ in combination‐treated mice (Figure 9l). These findings collectively demonstrated that LDRT +aPD‐L1 blockade through multiple mechanisms: enhancing CD8^+^ T cell tumor infiltration and cytolytic activity, suppressing Treg‐mediated immunosuppression and M2 type macrophage polarization, while potentiating pro‐inflammatory cytokine secretion. This multimodal immunomodulation effectively controls tumor progression and significantly extends survival in metastatic models.

## 3 Conclusion

This study proposes a strategy for addressing the dual challenges of tumor‐targeted α‐radionuclide delivery and immune synergy. The approach involves the innovative engineering of MOF(Fe)‐based self‐powered α radionuclide nanomedicine (^223^Ra‐MOF(Fe)@TPP). The nanodrug performs mitochondrial targeting, achieving synchronized TAT and CDT driven by a single radionuclide. The core mechanism involves α particle‐induced ionization to disrupt mitochondrial function and secondary electron‐activated MOF(Fe) catalysis, converting endogenous H_2_O_2_ into cytotoxic ·OH, while γ photons enable real‐time imaging. The experimental evidence demonstrated that ^223^Ra‐MOF(Fe)@TPP has the capacity to remodel the tumor microenvironment via three distinct mechanisms: namely, mitochondrial autophagy, PERK/eIF2α pathway‐mediated endoplasmic reticulum stress, and ICD. A rigorous and systematic validation process was undertaken on animal models to assess the efficacy of the drug in monotherapy for tumor killing, abscopal effects, metastasis suppression, and combinatorial immunotherapy. The proposed “radionuclide energy internal cycling” strategy integrates physical damage, chemical catalysis, and immune activation through a three‐tier synergy, providing a transformative solution to overcome traditional radiotherapy's physical‐biological barriers and immunosuppressive microenvironments. This work represents a groundbreaking advance in the field of precision radiodynamic immunotherapy at the organelle level.

## Conflict of Interest

The authors declare no conflict of interest.

## Supporting information



Supporting Information

## Data Availability

The data that support the findings of this study are openly available in 0 at 0, reference number 0.
