# Supplementary material for: Self‐Powered α Radionuclide Nanomedicine: Mitochondria‐Targeted Multimodal Energy Recycling for Amplified Radioimmunotherapy
Source: Adv Mater. 2025 Jul 2;37(37):2504612. doi: 10.1002/adma.202504612 (PMC12447027; doi:10.1002/adma.202504612)
Supplement: Supplementary file 1 — Supporting Information [file ADMA-37-2504612-s001.docx]

**Self-Powered α Radionuclide Nanomedicine:** **Mitochondria-Targeted Multimodal Energy Recycling for Amplified Radioimmunotherapy**

**Xian Li^#^, Chaochao Wang^#^, Yelin Wu, Jiajia Zhang, Han Zhang, Shanshan Qin, Linglin Tang, Fei Yu^*^**

# First authorship

* Corresponding Author

X. Li, J. Zhang, H. Zhang, S. Qin, F. Yu

Department of Nuclear Medicine, Shanghai Tenth People’s Hospital, Tongji University School of Medicine, Shanghai, 200072, P. R. China. Institute of Nuclear Medicine, Tongji University School of Medicine, Shanghai, 200072, P. R. China.

Email: 0910110013yufei@tongji.edu.cn

C. Wang, Y. Wu

Department of Medical Ultrasound, Shanghai Tenth People’s Hospital, School of Medicine, Tongji University, Shanghai, 200072 P. R. China

L. Tang

Department of Nuclear Medicine, Renji Hospital, School of Medicine, Shanghai Jiao Tong University, No. 160 Pujian Road, Shanghai, 200127, China

**4 Experimental Section**

**Materials and Reagents**
Ferric chloride hexahydrate (FeCl_3_·6H_2_O), 2-aminoterephthalic acid (NH_2_-BDC), 5,5-dimethyl-1-pyrroline N-oxide (DMPO), triphenylphosphine, and polyethylene glycol were purchased from Shanghai Aladdin Biochemical Technology Co., Ltd. (China). N, N-dimethylformamide (DMF) and hydrogen peroxide (H_2_O_2_) were supplied by Sinopharm Chemical Reagent Co., Ltd. (Shanghai, China).

The synthesis of NH_2_-MIL-88B was performed as follows: first, 0.541 g of FeCl_3_·6H_2_O and 0.362 g of NH_2_-BDC were dissolved in 42 mL DMF under continuous stirring at room temperature for 1 h. The mixture was transferred into a 100 mL Teflon-lined stainless-steel autoclave and subjected to solvothermal treatment at 120 °C for 12 h, followed by natural cooling to room temperature. The NH_2_-MIL-88B powder was collected by centrifugation at 8000 rpm for 5 min, washed repeatedly with DMF and ethanol, and dried at 70 °C for 12 h to obtain a brown powder.

Dissolve TPP (40 μmol) in 5 mL of PBS buffer (pH 5.5). Add EDC (25 mg, 480 μmol) and NHS (15 mg, 48 μmol) (molar ratio 1:1.2:1.2) and stir for 2 h at room temperature to generate active NHS ester. ^223^Ra-NH_2_-MIL-88B (5 mg) was added and dispersed by sonication for 10 min. Adjust the pH to 7.4 and stir at room temperature for 24 hours. Centrifugation, washing and drying yielded ^223^Ra-NH_2_-MIL-88B(Fe)@TPP. ^223^Ra-NH_2_- MIL-88B(Fe)@TPP was then added to PEG solution and stirred magnetically for 12 h at room temperature. Centrifugation, washing and drying for 24 hours gave the final product ^223^Ra-NH_2_-MIL-88B(Fe)@TPP-PEG

**Material Characterization**
The morphology and elemental distribution of MOF(Fe) nanoparticles were analyzed using transmission electron microscopy (TEM, JEOL 2011) coupled with energy-dispersive X-ray spectroscopy (EDS). Hydrodynamic diameter and zeta potential were measured using a Malvern Zetasizer Nano ZS90. X-ray photoelectron spectroscopy (XPS, Thermo Scientific K-Alpha) was employed to determine the chemical valence states of Fe. Crystal structure information was acquired via X-ray diffraction (XRD, Bruker D8 Advance, Germany). Electron spin resonance (ESR) spectra were recorded on a Bruker EMXplus-6/1 spectrometer to detect hydroxyl radical (·OH) generation. Fourier-transform infrared (FTIR) spectra were collected using a Thermo Nicolet iS20 spectrometer. The concentration of MOF(Fe) nanocatalysts was quantified via inductively coupled plasma optical emission spectroscopy (ICP-OES, Agilent 5110).

**Hydrodynamic diameter and colloidal stability Determination**

Hydrodynamic diameter and colloidal stability were determined by dynamic light scattering (DLS) using Zetasizer Nano ZS90. Measurements were performed in triplicate at 25 ℃ with samples dispersed in deionized water, PBS and 10% FBS. Results are reported as the mean hydrodynamic diameter ± standard deviation (nm) alongside the polydispersity index (PDI ± SD), which reflects particle size distribution homogeneity.

Surface charge (Zeta potential) was measured under identical conditions via electrophoretic light scattering and reported as the mean value ± standard deviation (mV).

**Detection of Fe^2+^ Generation**
Fe^2+^ was quantified using the phenanthroline (Phen) colorimetric method. Phen forms an orange-red [Fe (Phen)_3_]^2+^ complex with Fe^2+^, exhibiting a characteristic absorption peak at 510 nm. A Phen solution (25 μg/mL) was prepared, and 50 μg/mL of MOF(Fe) or 50 nCi of ^223^Ra-MOF(Fe) was added. Absorbance spectra (400–800 nm) were recorded at different time points using a UV-vis spectrophotometer. Dose-and concentration-dependent effects were further analyzed.

**Detection of Hydroxyl Radical (·OH) Generation**
·OH production was confirmed by ESR spectroscopy using DMPO as a spin-trapping agent. Reaction mixtures containing 40 μL of DMPO buffer were prepared in dark Eppendorf tubes as follows: (I) Control (H_2_O); (II) MOF(Fe); (III) ^223^Ra-MOF(Fe); (IV) H_2_O_2_; (V) H_2_O_2_ + MOF(Fe) (pH 6.5); (VI) H_2_O_2_ + ^223^Ra-MOF(Fe)(pH 6.5); (VII) H_2_O_2_ + MOF(Fe) (pH 4.5); (VIII) H_2_O_2_ + ^223^Ra-MOF(Fe) (pH 4.5). Final concentrations of DMPO, H_2_O_2_, MOF(Fe), and ^223^Ra-MOF(Fe) were 1 mM, 5 mM, 50 μg/mL, and 50 nCi/mL, respectively. After 30 min, the mixture was loaded into quartz capillaries, and ESR spectra were recorded on a Bruker EMX-8/2.7 spectrometer under the following conditions: microwave frequency = 9.872 GHz, microwave power = 6.375 mW, modulation frequency = 100.00 kHz, modulation amplitude = 1.00 G.

**Peroxidase-Mimicking Activity Assay**
The peroxidase-like activity of MOF(Fe)@TPP, ^223^Ra-MOF(Fe), and ^223^Ra-MOF(Fe)@TPP was evaluated using 3,3’,5,5’-tetramethylbenzidine (TMB) as a chromogenic substrate. The catalytic reaction system contained 30 nCi of material and 500 μM H_2_O_2_ in pH 4.5 acetate buffer (0.01 M). After adding 1 mM TMB, absorbance spectra (200–900 nm) were recorded using a Shimadzu UV-2550 spectrophotometer, focusing on the characteristic peak at 652 nm. Control experiments were performed with H_2_O_2_ and TMB only. To investigate catalytic kinetics, experiments were repeated at pH 4.5, 5.5, and 6.5. The Michaelis-Menten equation was used to calculate Km (substrate affinity) and Vmax (maximum reaction rate), with initial reaction rates (ν) determined using ν = ΔA/ (Δt × ε × l), where ε = 39,000 M^-1^ cm^-1^ (molar extinction coefficient of oxidized TMB) and l = 1 cm (path length).

**Cellular Uptake Assay**
MC38 cells were seeded in 12-well plates (1 × 10^5^ cells/mL/well) and cultured for 24 h. Cells were then treated with media containing 30 nCi of MOF(Fe)@TPP, ^223^Ra-MOF(Fe), or ^223^Ra-MOF(Fe)@TPP. At specified time points (0.5–72 h), cells were washed with cold PBS and lysed with 1 M NaOH. Radioactivity was quantified using a γ-counter, and non-radioactive components were analyzed via inductively coupled plasma mass spectrometry (ICP-MS) to track intracellular iron levels. Data were cross-validated through dual measurements of radioactivity and elemental mass concentration.

**In Vitro Cytotoxicity Assay**
MC38 cells (5 × 10^3^ cells/well) were seeded in 96-well plates and treated with MOF(Fe)@TPP (0-320 μg/mL), ^223^RaCl_2_, ^223^Ra-MOF(Fe), or ^223^Ra-MOF(Fe)@TPP (0–200 nCi). After 24 h, CCK-8 reagent (10 μL/well) was added, and absorbance at 450 nm was measured using a BioTek Synergy H1 microplate reader. Cell viability was calculated relative to untreated controls.

**Live/Dead Cell Staining and Apoptosis Analysis**
Treated cells were stained with 2 μM Calcein-AM (live cells, 488/517 nm) and 4 μM PI (dead cells, 543/617 nm) for 15 min. Fluorescence images were captured using a Leica DMi8 microscope, and viability ratios were calculated via ImageJ. After 24 h of treatment, MC38 cells were trypsinized, centrifuged (1000 rpm, 5 min), and resuspended in binding buffer. Cells were stained with FITC-Annexin V and PI (5 μL and 2 μL, respectively) for 15 min in the dark. Apoptotic populations (early: FITC⁺/PI⁻; late: FITC⁺/PI⁺) were analyzed using a BD FACSCalibur flow cytometer and FlowJo software.

**Intracellular ROS Detection and ATP Content Measurement**
MC38 cells (1 × 10^6^ cells/well) in 6-well plates were treated for 24 h and incubated with 10 μM DCFH-DA for 30 min. Fluorescence intensity (488/530 nm) was quantified via microscopy and flow cytometry (BD FACSCalibur). Untreated cells served as background controls. ATP levels in treated cells were measured using a luciferase-based kit. Luminescence was recorded, and data were normalized to protein concentration.

**Mitochondrial Membrane Potential Assay**

Cells (2 × 10^5^ cells/well) were stained with 2 μM JC-1 at 37°C, 5% CO_2_ protected from light for 30 min. Flow Cytometry: Cells were analyzed using a flow cytometer with FITC (monomer, green) and PE (aggregates, red) channels. The red-to-green fluorescence ratio (red/green Ratio) of the cell population was calculated using FlowJo software. Immunofluorescence Imaging: green (530 nm, monomer) and red (590 nm, aggregates) fluorescence images were captured using a Nikon Ti2 microscope. The red-to-green fluorescence ratio within individual cells or regions was calculated using image analysis software.
**Western Blotting**

To evaluate the ability of ^223^Ra-MOF(Fe)@TPP to induce both mitophagy and endoplasmic reticulum (ER) stress, the expression levels of key autophagy markers (LC3, p62, PINK1, Parkin) and ER stress markers (CHOP, p-PERK, PERK, p-eIF2α, eIF2α,) were analyzed by Western blotting. MC38 cells were treated for 24 hours with PBS, ^223^RaCl_2_ (50 nCi/mL), MOF(Fe)@TPP (100 μg/mL), ^223^Ra-MOF(Fe) (50 nCi/mL), ^223^Ra-MOF(Fe)@TPP (50 nCi/mL), or chloroquine (CQ, 10 μM, as a positive control autophagy inhibitor) or Sodium 4-phenylbutyrate (4-PBA sodium, ER stress inhibitor). Following treatment, cells were harvested and lysed to obtain total protein. The protein lysate was immunoprecipitated, resolved by SDS-PAGE using 12% separating gels, and transferred onto PVDF membranes. Membranes were probed with primary antibodies against LC3 (1:1000, Abmart, #T55992), p62 (1:1000, Abmart, #T55546), PINK1 (1:1000, Proteintech, #23274-1-AP), Parkin (1:1000, Proteintech, #14060-1-AP), CHOP (1:1000, Abmart, #[Catalog number missing]), p-eIF2α (1:1000, Abmart, #TA3087), eIF2α (1:1000, Abmart, #PS13083S), p-PERK (1:1000, Abmart, #PS00157), PERK (1:1000, Abmart, #MA8131), along with loading control antibodies for β-actin (1:5000, Abmart,#P30002), β-tubulin (1:5000, Abmart, #R20005), and GAPDH (1:5000, Abmart, #P60037). Protein bands were visualized using an enhanced chemiluminescence (ECL) detection system. Band intensities were quantified using ImageJ software to determine the LC3-II/β-actin ratio, p62 levels, PINK1 levels, Parkin levels, LC3-II/β-tubulin ratio, CHOP levels, p-eIF2α levels, eIF2α levels, p-PERK levels, and PERK levels for evaluating the activation of both autophagic flux and ER stress.

**CRT/HMGB1 Immunofluorescence**
Cells were fixed, permeabilized, and blocked before incubation with primary antibodies Calreticulin(1:500, ABclonal, A20986) HMGB1(1:100, ABclonal, A16002), and Alexa Fluor-conjugated secondary antibodies (1:500). Nuclei were stained with DAPI, and images were acquired using confocal microscopy. To detect the expression and localization of CRT and HMGB1 in tumor tissues, cells were fixed in 4% paraformaldehyde for 15 minutes and permeabilized with 0.1% Triton X-100 for 10 minutes. CRT was labeled using a rabbit anti-CRT antibody (1:1000), while HMGB1 was labeled using a mouse anti-HMGB1 antibody (1:1000). The corresponding secondary antibodies were Alexa Fluor 594-conjugated goat anti-rabbit IgG for CRT and Alexa Fluor 594-conjugated goat anti-mouse IgG for HMGB1 (both used at 1:500 dilution). Cell nuclei were counterstained with DAPI (1 μg/mL) for 5 minutes. Fluorescent signals were captured using a confocal microscope, and the images were analyzed using ZEN software.

**In Vivo Imaging and Biodistribution**

Tumor-bearing mice were randomly divided into two groups: one for intravenous injection and one for intratumoral injection. Each group was injected with 500 nCi of the ^223^Ra-MOF(Fe)@TPP complex. Whole-body imaging of the mice was then performed at various time points (e.g., 4, 24, 48, 96, 168, and 336 hours) using a SPECT/CT scanner to determine the distribution of the radiopharmaceuticals in the tumors and non-target organs (e.g., the liver, spleen, and bone). Image analysis software was used to quantitatively calculate the intensity of radioactivity in the tumor region to assess drug retention. Simultaneously, a γ counter was used to perform quantitative radioactivity analysis of the tumor and key organs (e.g., liver, spleen, and bones) to further verify the enrichment of the drug in the tumor region and its uptake in non-target organs.

**In Vivo Antitumor Evaluation**

MC38 tumor-bearing mice (n=6/group) were treated with PBS, MOF(Fe)@TPP, ^223^RaCl_2_ (900 nCi), ^223^Ra-MOF(Fe), or ^223^Ra-MOF(Fe)@TPP. During treatment, the tumor volume (V = L × W^2^/2)was monitored for 14 days, and the body weight changes of the mice in each group were recorded to assess systemic toxicity. The mice were executed at the endpoint of treatment, and tumor tissues were taken for photographic recordings to compare the differences in tumor volume between the groups. Meanwhile, blood samples were collected for routine blood analysis and renal function indexes (e.g., blood creatinine, urea nitrogen) to assess the effects of the drugs on systemic functions. Major organs (heart, liver, spleen, lungs and kidneys) were taken for histological evaluation, and the histopathological changes of each organ were observed by H&E staining. In addition, tumor tissues were taken for H&E staining and TUNEL fluorescence staining to assess the necrotic areas and apoptosis of tumor cells, respectively.

**Tumor Immune Cell Profiling**
Spleens and tumors were dissociated into single-cell suspensions, stained with multicolor antibodies: APC anti-mouse CD45 antibody, FITC anti-mouse CD3 antibody, BV421 anti-mouse CD4, APC/Cy7 anti-mouse CD8a antibody, BV605 anti-mouse CD25 antibody, PE anti-mouse Foxp3 antibody, PE/Cy7 anti-mouse CD45 antibody, FITC anti-mouse CD11c antibody, APC anti-mouse CD80 antibody, PE anti-mouse CD86 antibody (Biolegend) and analyzed via flow cytometry. Cytokine levels (IL-10, IFN-γ, IL-12p70, TNF-α, Elabscience ) were quantified using ELISA.

**PD-L1 Antibody Combination Therapy**

The MC38 mouse bilateral tumor model was constructed and observed until the primary tumor reached a size of about 100 mm^3^. The tumor-bearing mice were randomly divided into 6 groups(n=4): a control group; aPD-L1 monotherapy (200 μg/mouse), ^223^Ra-MOF(Fe), ^223^Ra-MOF(Fe)+aPD-L1, ^223^Ra-MOF(Fe)@TPP, ^223^Ra-MOF(Fe)@TPP+aPD-L1. Primary tumors received an intratumoral injection of 900 nCi of ^223^Ra-MOF(Fe) or ^223^Ra-MOF(Fe)@TPP while left distal tumors remained untreated. The aPD-L1 antibody was administered via intraperitoneal injection at a dose of 75 μg per mouse on days 1, 3, and 5. The growth of primary and distant tumors and body weight were monitored every other day.

**Data Analysis**
Data are presented as mean ± SD. Statistical analysis was performed using GraphPad Prism 9.0. One-way ANOVA (multiple groups) or two-tailed t-test (two groups) was applied. Significance levels: **p* < 0.05, ***p* < 0.01, ****p* < 0.001. All experiments were independently repeated three times.


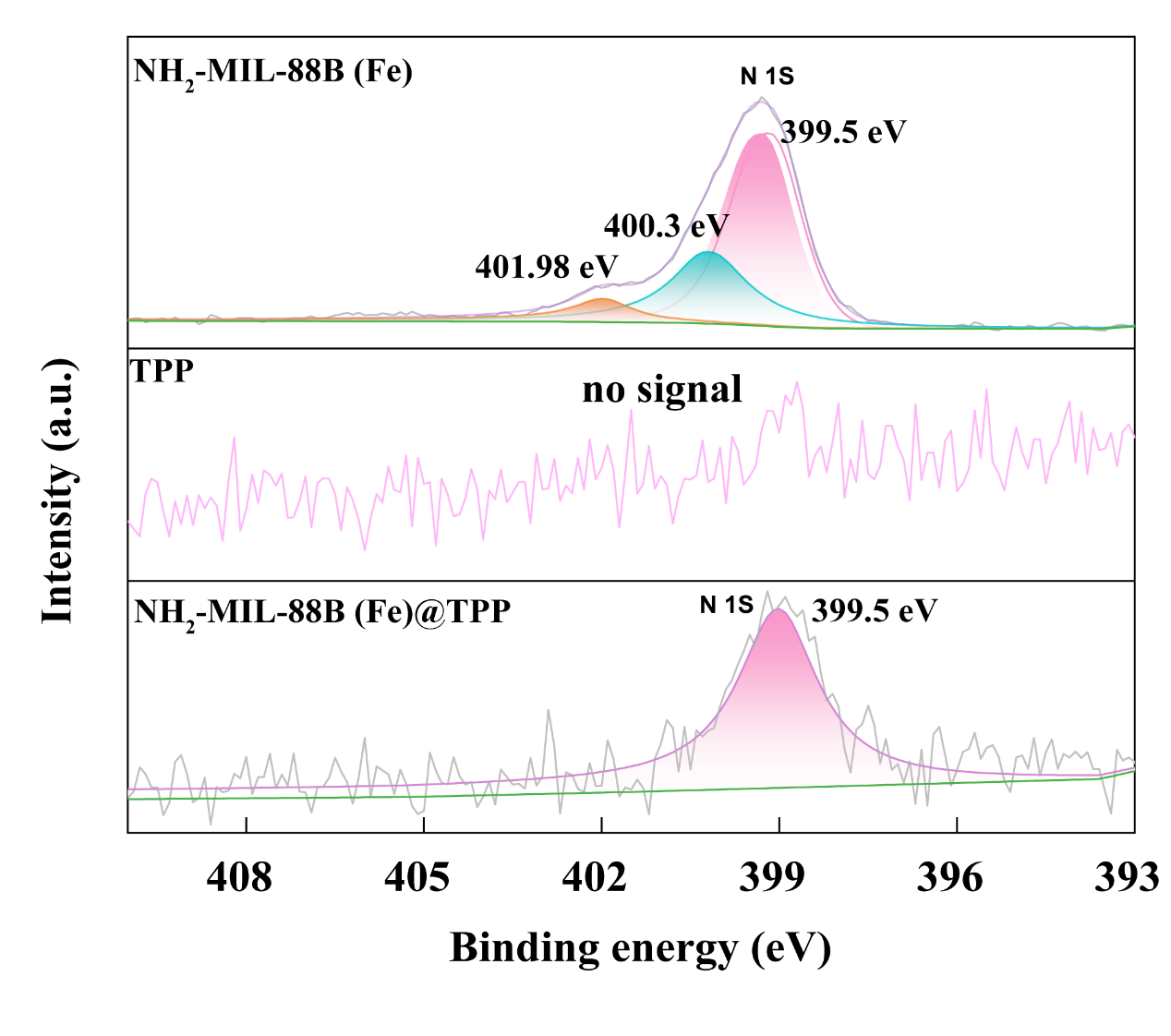


**Figure S1**. XPS spectra of NH_2_-MIL-88B (Fe), TPP and NH_2_-MIL-88B (Fe)@TPP.


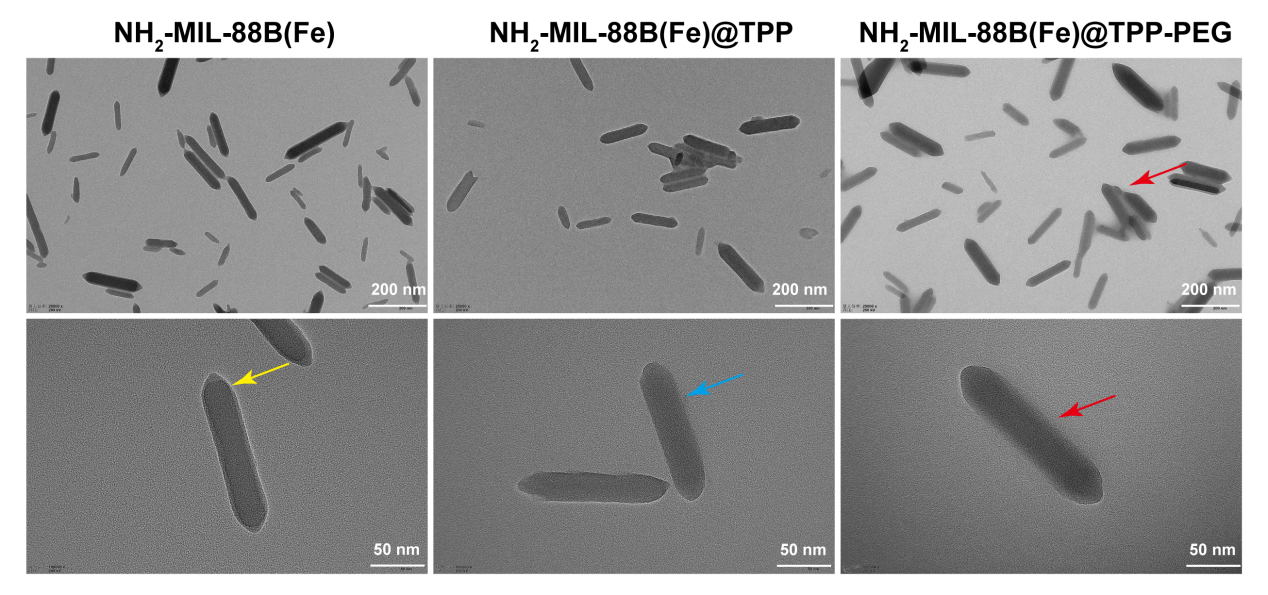


**Figure S2** TEM images of NH_2_-MIL-88B(Fe), NH_2_-MIL-88(Fe)@TPP and NH_2_-MIL-88(Fe)@TPP-PEG nanocatalysts. Scale bar, 200 nm, 50nm.

The parent NH_2_-MIL-88B(Fe) has a regular fusiform shape with sharp edges and well-defined boundaries (yellow arrow). TPP modification leads to partial of NH_2_-MIL-88(Fe)@TPP aggregation and surface roughness (blue arrow). NH_2_-MIL-88B(Fe)@TPP-PEG has enhanced edge blurring (red arrow).


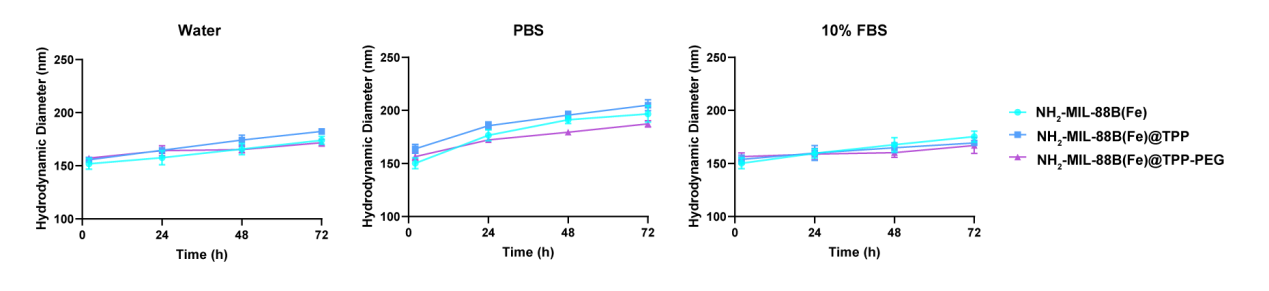


**Figure S3** Long-term stability of hydrodynamic diameters of NH_2_-MIL-88B(Fe), NH_2_-MIL-88(Fe)@TPP, NH_2_-MIL-88(Fe)@TPP-PEG in different media (n = 3). Data are expressed as mean ± SD. **p* < 0.05, ***p* < 0.01, ****p* < 0.001.

.


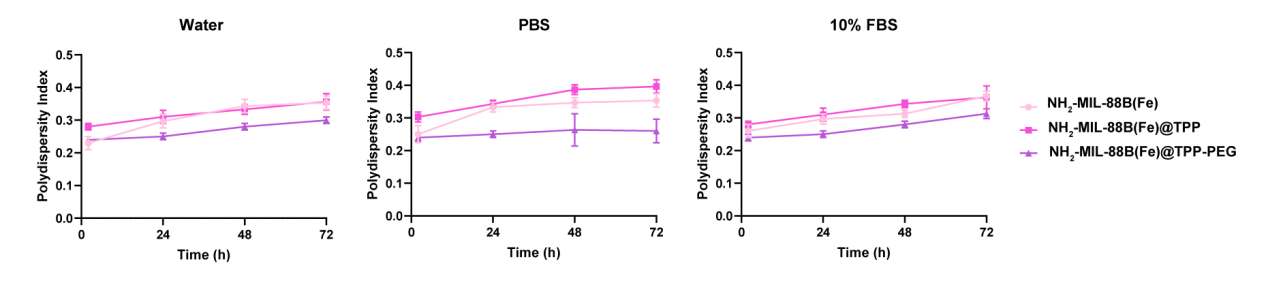


**Figure S4**

Long-term stability of polymer dispersity index of NH_2_-MIL-88B(Fe), NH_2_-MIL-88(Fe)@TPP, NH_2_-MIL-88(Fe)@TPP-PEG in different media (n = 3). Data are expressed as mean ± SD. **p* < 0.05, ***p* < 0.01, ****p* < 0.001.


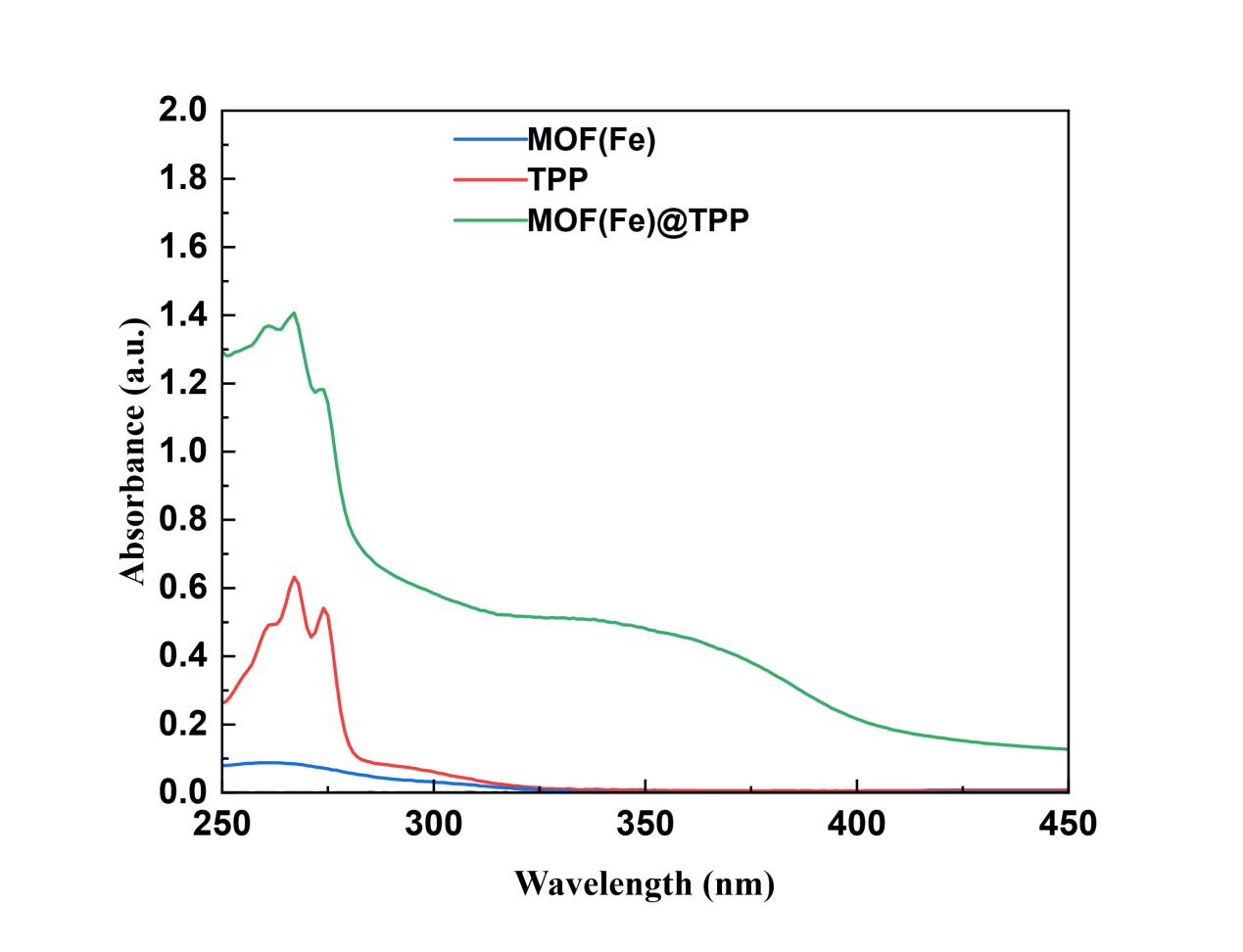


**Figure S5**. The absorptance spectrum of MOF(Fe), TPP and MOF(Fe)@TPP NPs.

**Figure S6**. Relative Fe concentration of MC38 cells treated with ^223^Ra-MOF(Fe) and ^223^Ra-MOF(Fe) at different time (n = 3). Data are expressed as mean ± SD. **p* < 0.05, ***p* < 0.01, ****p* < 0.001.

**Figure S7**. Quantitative data of average mitochondrial diameter(n = 3) and mitochondria number (n = 30) under different treatments. Data are expressed as mean ± SD. **p* < 0.05, ***p* < 0.01, ****p* < 0.001.

**Figure S8**. Quantitative data of intracellular ROS of MC38 cells under different treatments using FCM analysis(n = 3). Data are expressed as mean ± SD. **p* < 0.05, ***p* < 0.01, ****p* < 0.001.


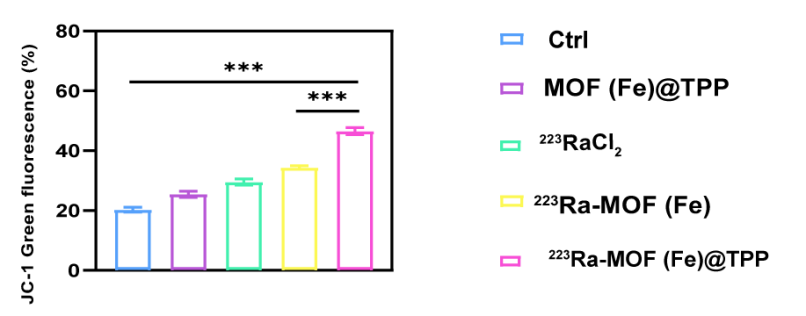


**Figure S9**. Quantitative data of mitochondrial membrane potential assessment of MC38 cells under different treatments using FCM analysis(n = 3). Data are expressed as mean ± SD. **p* < 0.05, ***p* < 0.01, ****p* < 0.001.


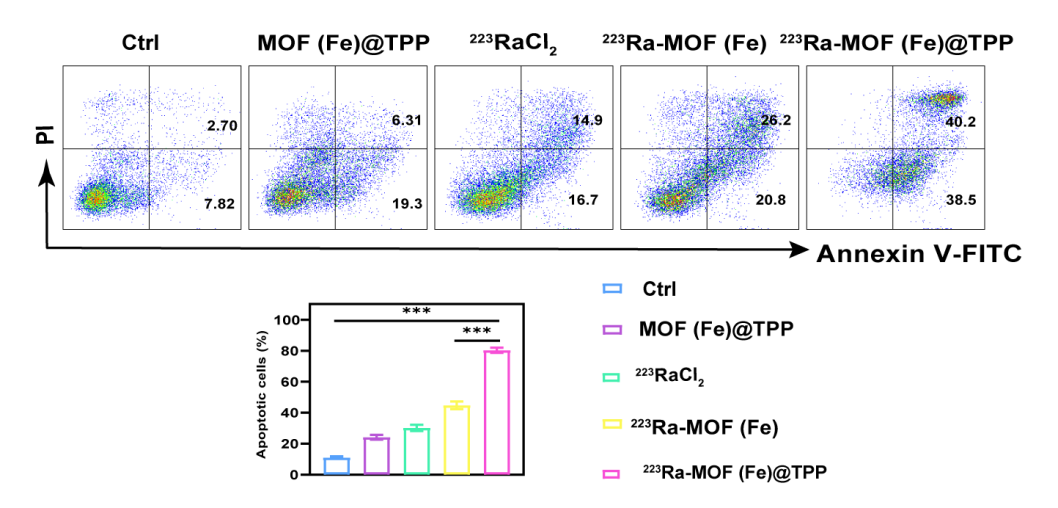


**Figure S10**. Apoptosis assessment of MC38 cells under different treatments using FCM analysis, along with quantification data of dead cells, with quantification of apoptotic cells of FCM analysis (n = 3). Data are expressed as mean ± SD. **p* < 0.05, ***p* < 0.01, ****p* < 0.001.


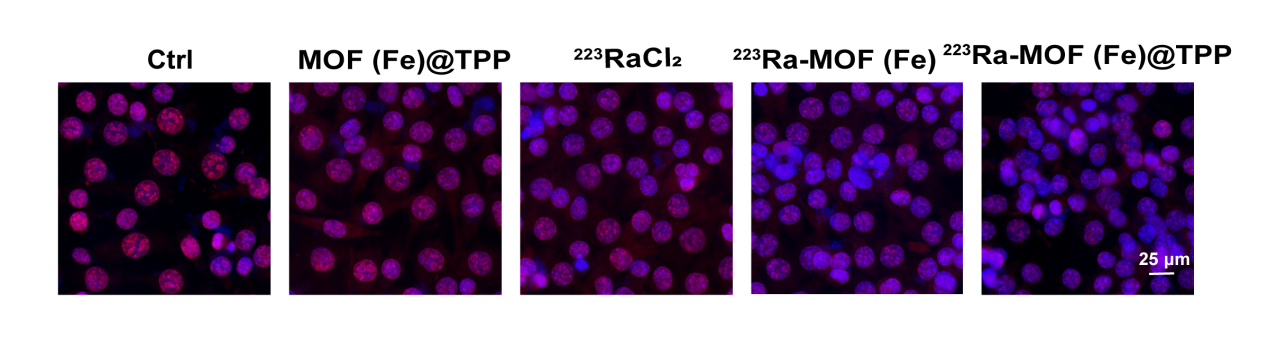


**Figure S11**. Evaluation of Ki-67 after different treatment. CLSM images of Ki-67 at tumor site in MC38 tumor-bearing mice after different treatments. Red fluorescence indicated the Ki-67 signal, and blue fluorescence showed the nucleus. Scale bar, 25 μm.


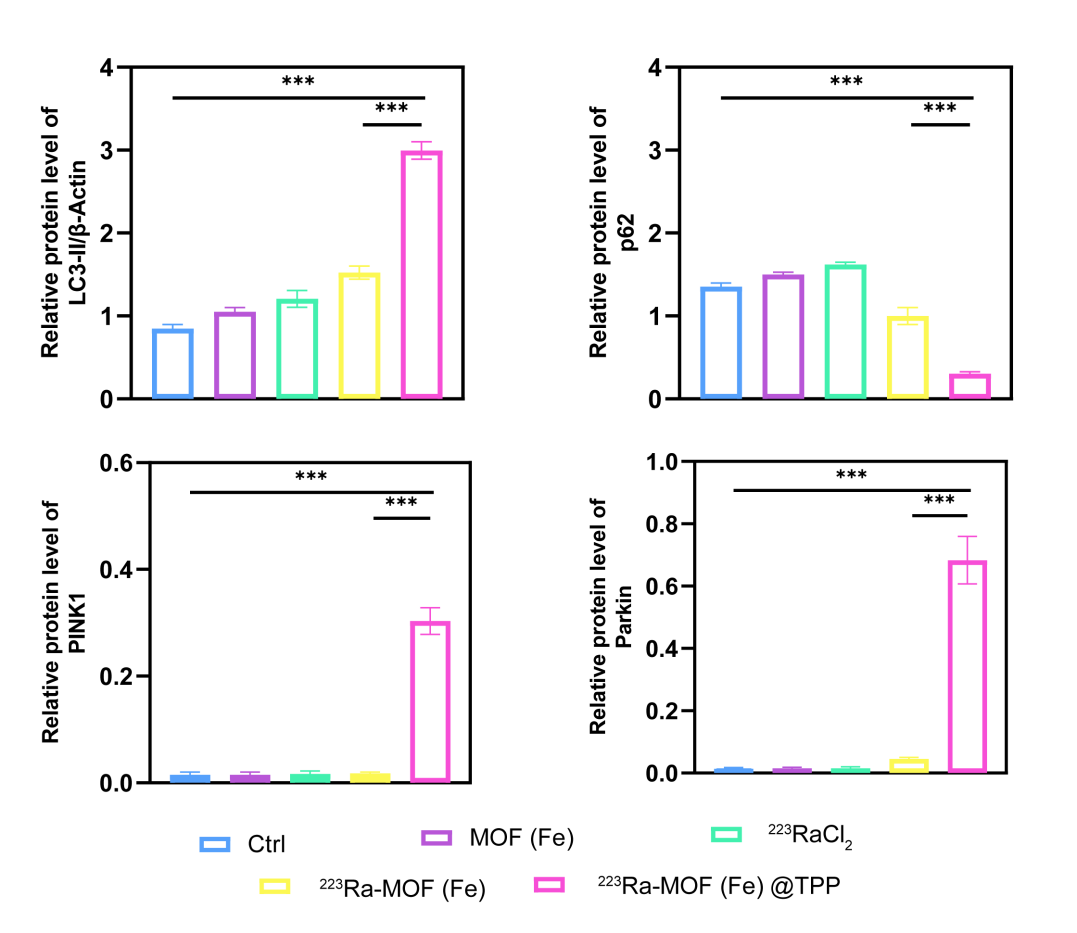


**Figure S12**. Quantitative analysis of the ratios of LC3 (LC3-I and LC3-II), p62, PINK1, Parkin expression levels determined by ImageJ, based on the typical images in Figure 3k (n = 3). Data are expressed as mean ± SD. **p* < 0.05, ***p* < 0.01, ****p* < 0.001.


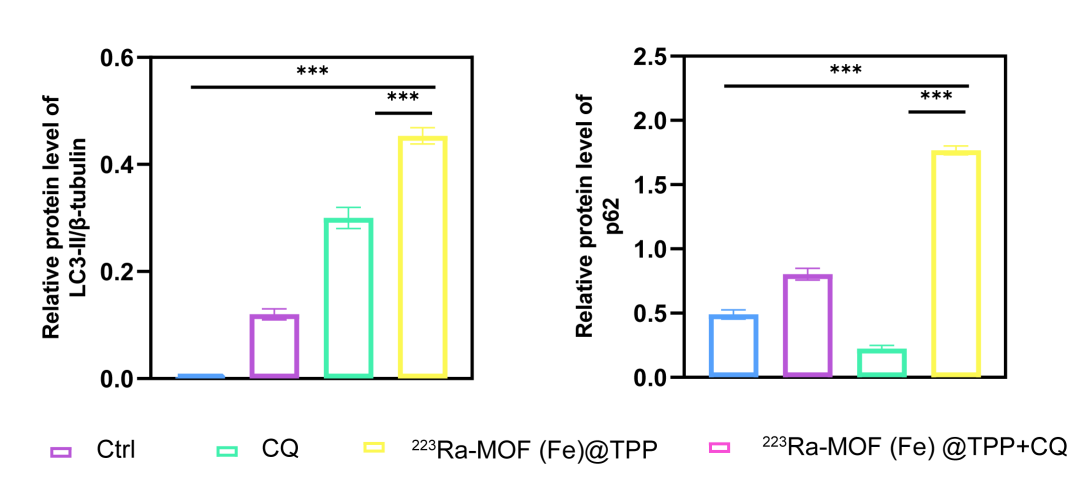


**Figure S13**. Quantitative analysis of the ratios of LC3 (LC3-I and LC3-II) and p62, expression levels determined by ImageJ, based on the typical images in Figure 3m (n=3). Data are expressed as mean ± SD. **p* < 0.05, ***p* < 0.01, ****p* < 0.001.


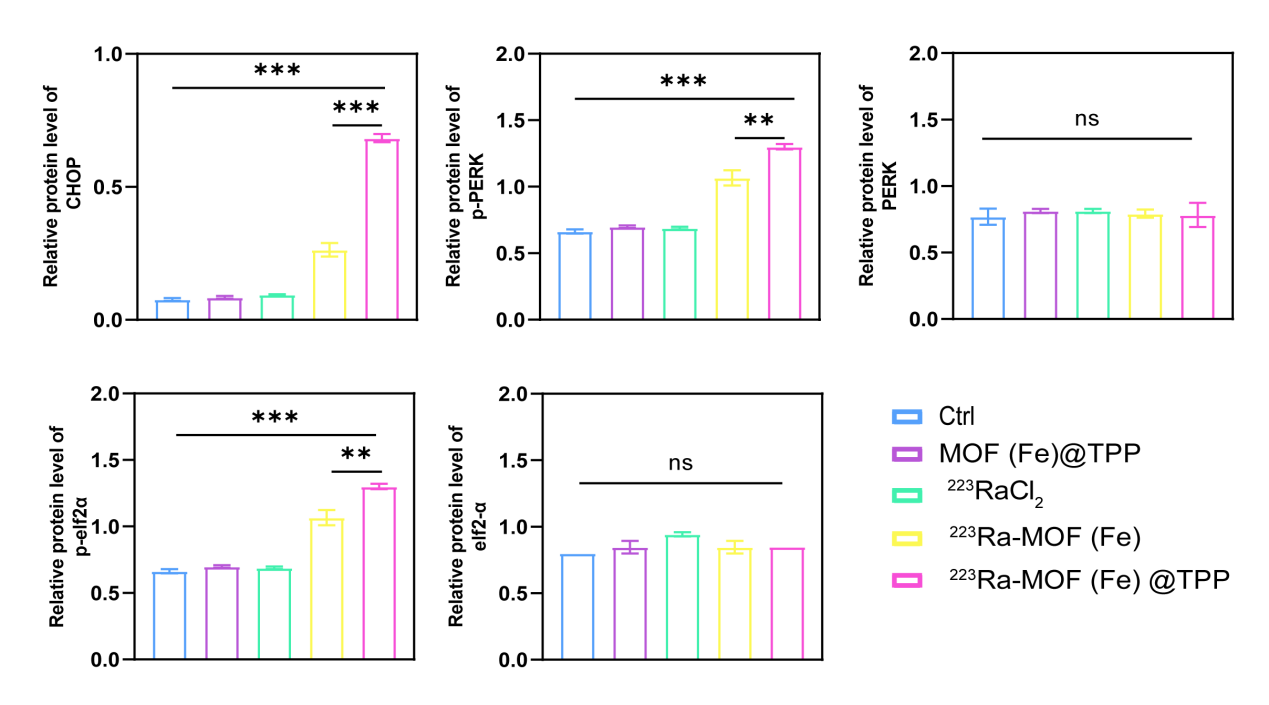


**Figure S14**. Quantitative analysis of CHOP, p-PERK, PERK, p-eIF2α and eIF2α determined by ImageJ, based on the typical images in Figure 4d (n = 3). Data are expressed as mean ± SD. **p* < 0.05, ***p* < 0.01, ****p* < 0.001.


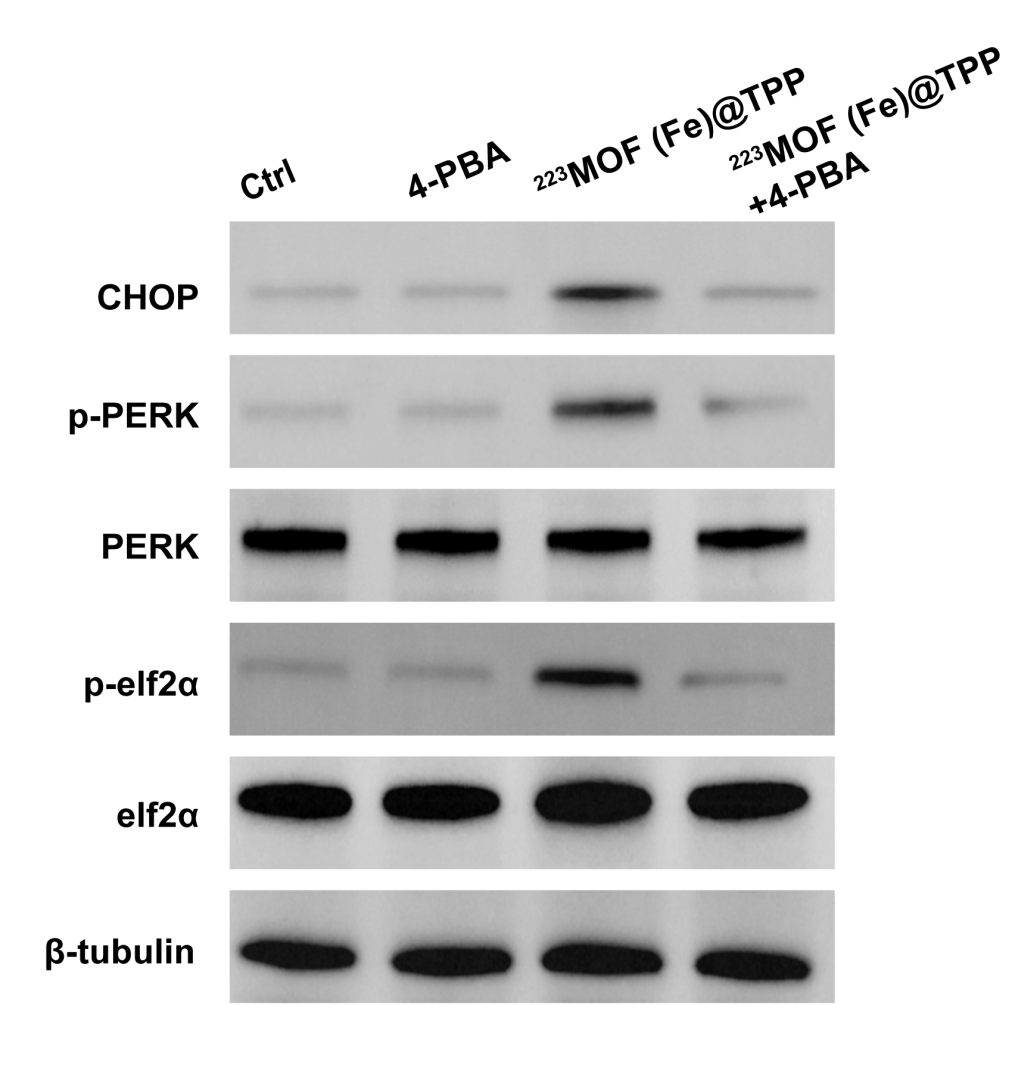


**Figure S15**. Representative western blot of indicated proteins in MC38 cells after different treatment.


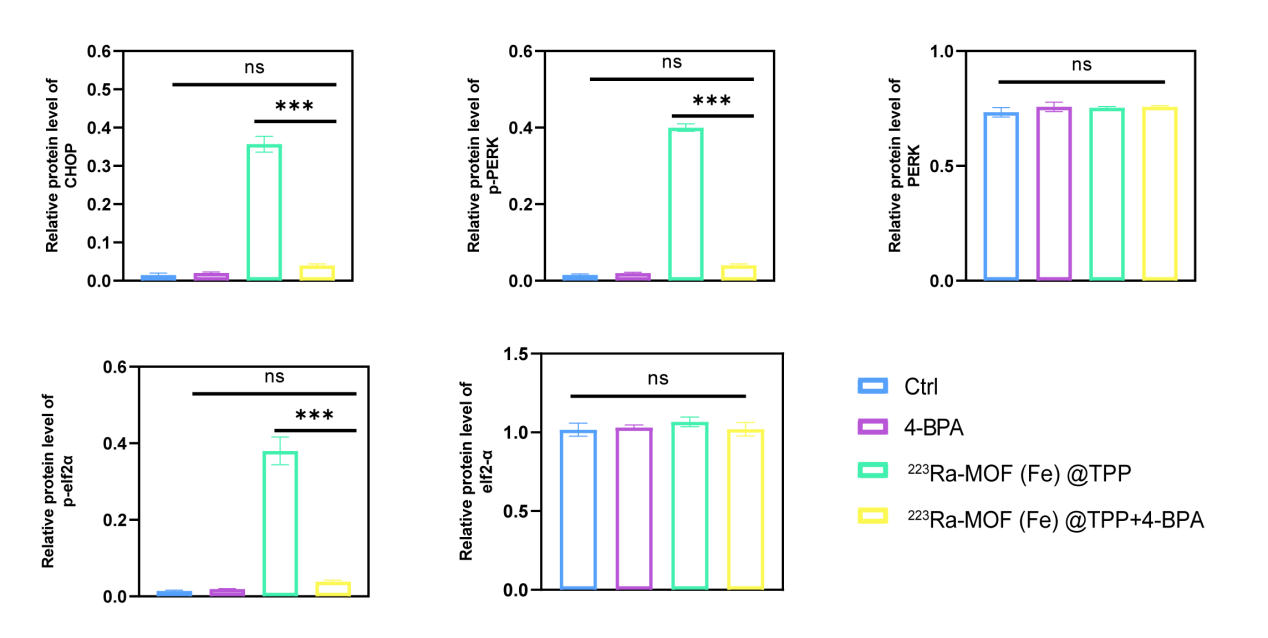


**Figure S16**. Quantitative analysis of CHOP, p-PERK, PERK, p-eIF2α and eIF2α determined by ImageJ, based on the typical images in Figure S15 (n = 3). Data are expressed as mean ± SD. **p* < 0.05, ***p* < 0.01, ****p* < 0.001.


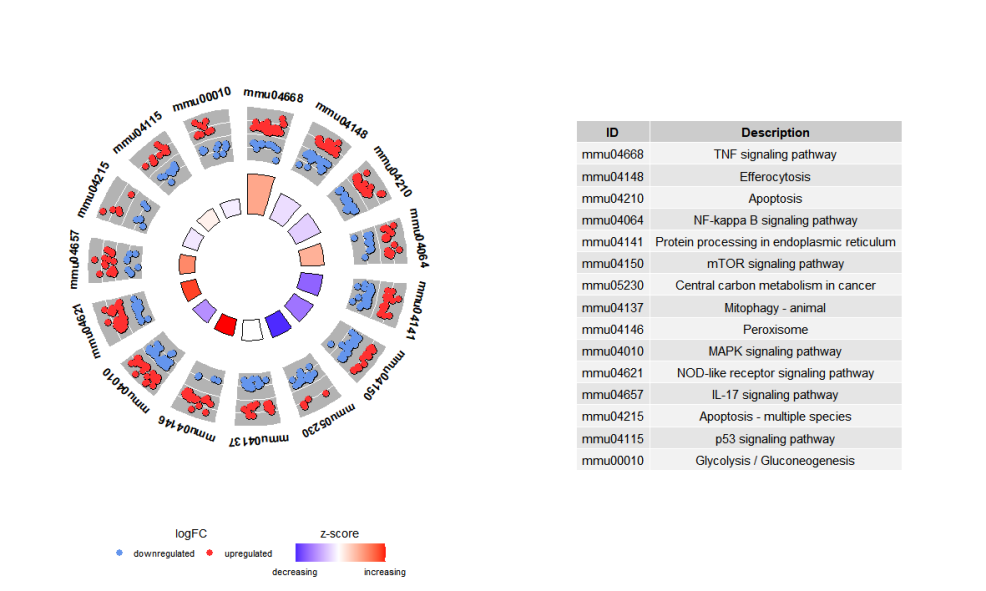


**Figure S17**. KEGG enrichment analysis for DEGs induced by ^223^Ra-MOF(Fe)@TPP treatment.


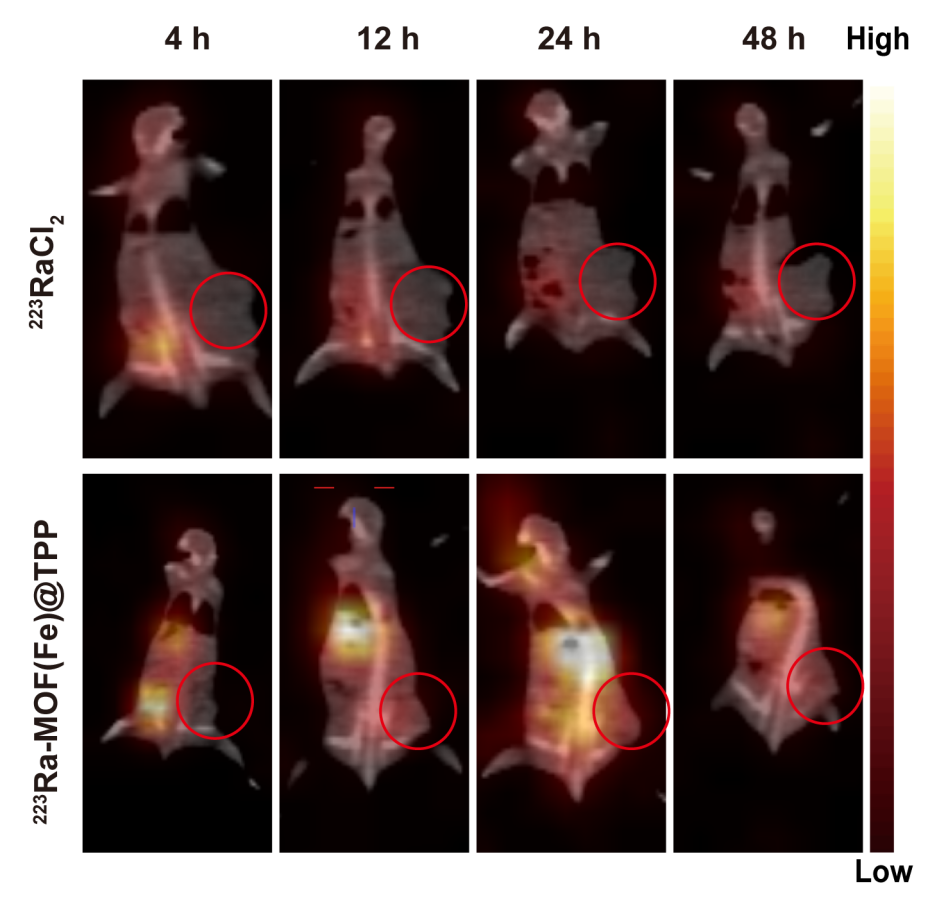


**Figure S18.** SPECT/CT images of MC38 tumor-bearing mice following injection intravenous of free ^223^RaCl_2_ and ^223^Ra-MOF(Fe)@TPP at 4 h, 12 h, 24 h, 48h.

**Figure S19**. Biodistribution of free ^223^RaCl_2_ (a) and ^223^Ra-MOF(Fe)@TPP (b) in mice at 4 h, 8 h and 24 h through intratumoral injection (n = 3).

**Figure S20**. Biodistribution of free ^223^RaCl_2_ (a) and ^223^Ra-MOF(Fe)@TPP (b) in mice at 4 h, 8 h and 24 h via intravenous injection (n = 3).

**Figure S21**. Biochemical analysis of mice blood 14 days after different treatment (n=3). (G1: Ctrl group, G2: MOF(Fe)@TPP, G3: ^223^RaCl_2_, G4: ^223^Ra-MOF(Fe), G5: ^23^Ra-MOF(Fe)@TPP). Data are expressed as mean ± SD. **p* < 0.05, ***p* < 0.01, ****p* < 0.001.

**Figure S22**. Blood biochemical analysis of mice blood 14 days after different treatment (n=3). (G1: Ctrl group, G2: MOF(Fe)@TPP, G3: ^223^RaCl_2_, G4: ^223^Ra-MOF(Fe), G5: ^23^Ra-MOF(Fe)@TPP). Data are expressed as mean ± SD. **p* < 0.05, ***p* < 0.01, ****p* < 0.001.


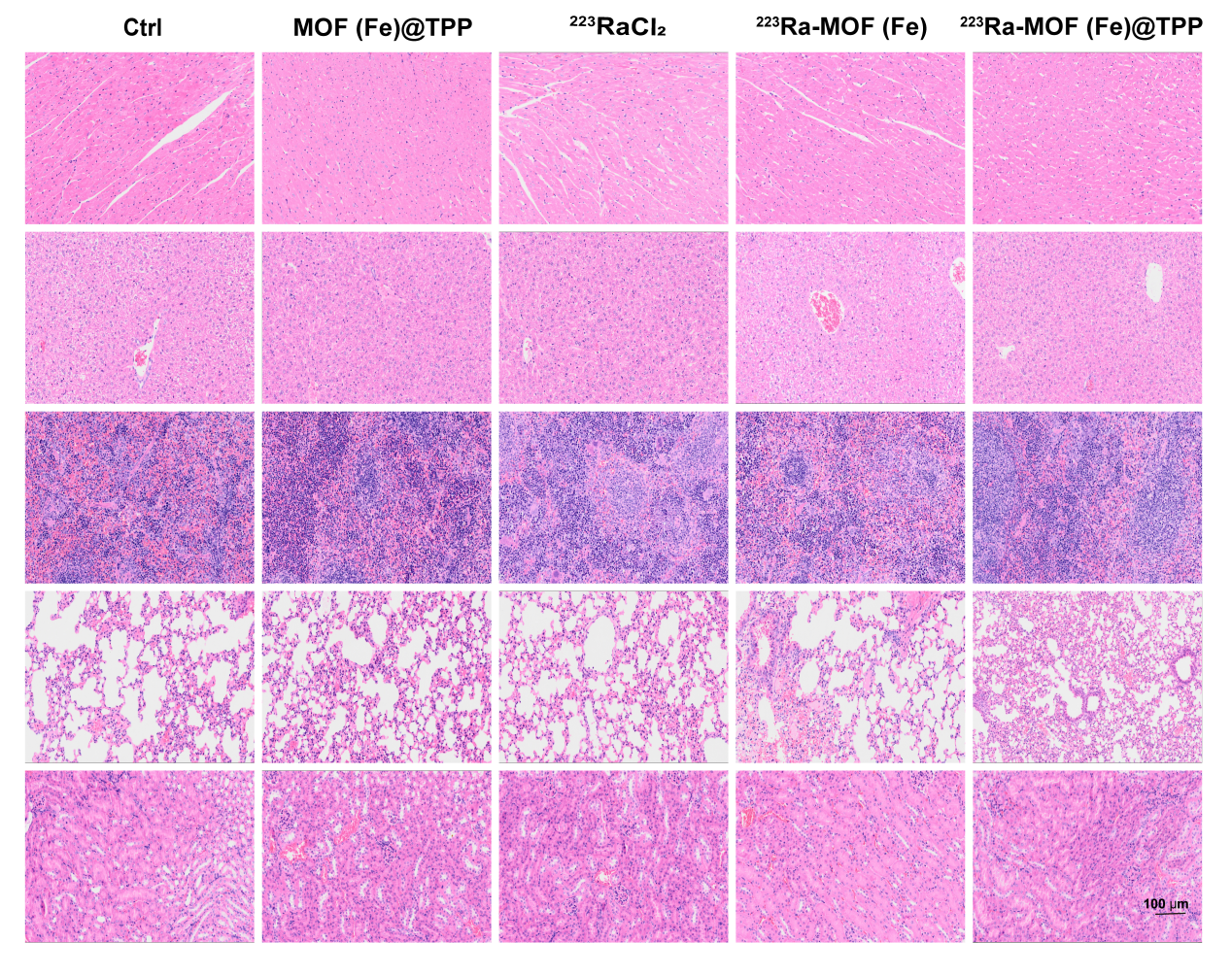


**Figure S23**. In vivo biosafety evaluation of different formulations. H&E staining of the major tissues (heart, liver, spleen, lung and kidney) collected at the end of treatment course. Scale bar: 100 μm.


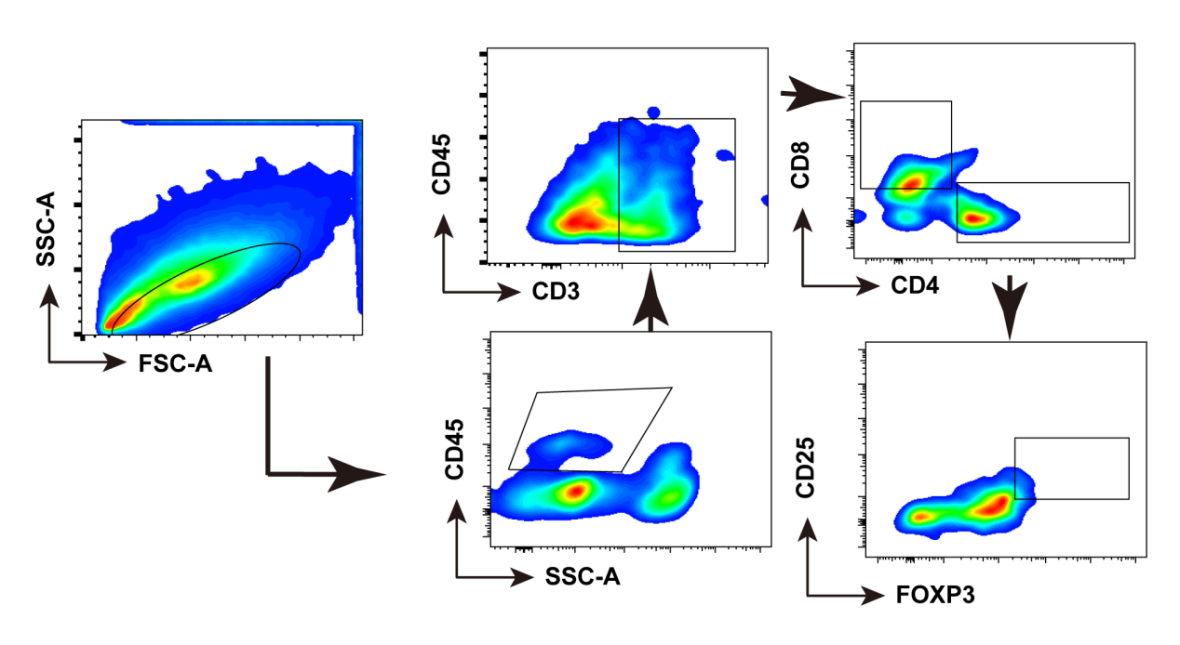


**Figure S24**. Flow cytometry sorting plots of intratumoral CTLs (CD45^+^CD3^+^CD8^+^) and Treg (CD45^+^CD4^+^CD25^+^Foxp3^+^) cells in MC38 tumor-bearing mice after receiving different treatments.


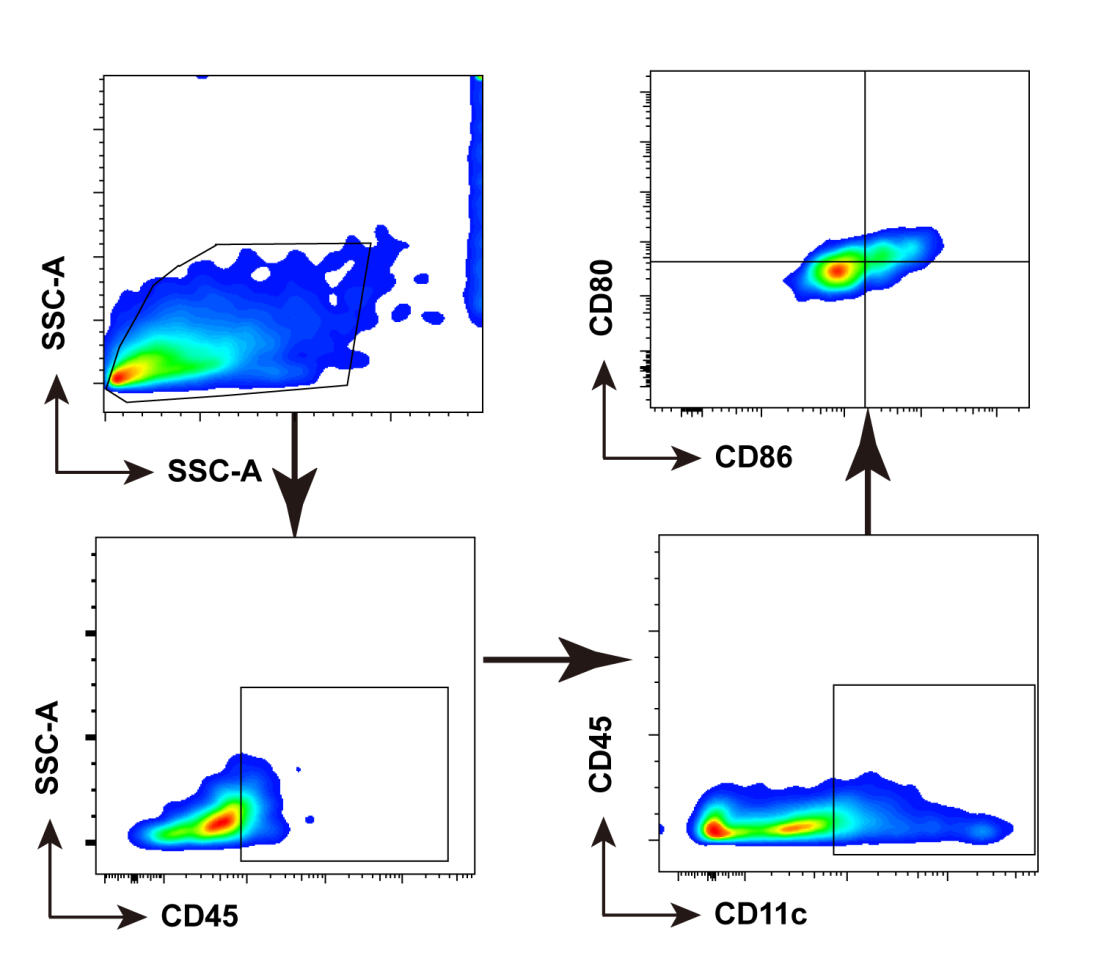


**Figure S25**. Flow cytometry sorting plots of DC maturation (CD45^+^CD11C^+^CD80^+^CD86^+^) in MC38 tumor after receiving different treatments.

**Figure S26**. In vivo biosafety evaluation of different formulations. Average body weight of the mice after receiving different treatments (n = 4). **p* < 0.05, ***p* < 0.01, ****p* < 0.001.

**Figure S27**. In vivo biosafety evaluation of different formulations. Average body weight of the mice after treatment (n = 5). **p* < 0.05, ***p* < 0.01, ****p* < 0.001.
